# Angiogenesis inhibitors and the risk of peripheral artery aneurysm and dissection: a cross-database study

**DOI:** 10.3389/fphar.2026.1932218

**Published:** 2026-09-22

**Authors:** Kaixuan Zhang, Qingsheng Lu

**Affiliations:** Division of Vascular Surgery, Department of General Surgery, Changhai Hospital, Naval Medical University, Shanghai, China

**Keywords:** angiogenesis inhibitors, FAERS, JADER, peripheral artery aneurysm and dissection, VEGF

## Abstract

**Background:**

Anti-angiogenic inhibitors (AI) have transformed cancer treatment but are associated with vascular toxicities. The specific association with peripheral artery aneurysm and dissection (PAAD) remains uncharacterized. This study aimed to evaluate the association between AI exposure and PAAD risk and to delineate the temporal patterns of these adverse events across different exposure windows.

**Methods:**

This analysis was conducted using the U.S. FDA Adverse Event Reporting System and the Japanese Adverse Drug Event Report databases. Disproportionality analysis using the Reporting Odds Ratio was applied to identify safety signals. Univariate and multivariate logistic regression models were fitted to detect associated factors and quantify the effects of distinct exposure duration windows. Kaplan-Meier analysis and the Weibull distribution were utilized to characterize time-to-onset patterns.

**Results:**

In total, 757,900 FAERS and 136,170 JADER reports were included. Disproportionality analyses identified significantly higher RORs for PAAD (ROR = 3.276 in FAERS; ROR = 8.465 in JADER) and peripheral artery dissection (PAD) (ROR = 6.275 in FAERS; ROR = 9.372 in JADER). The peripheral arterial aneurysm (PAA) signal was marginally non-significant in FAERS but significant in JADER (ROR = 8.113). Subtype-specific analyses showed small-molecule VEGFR-targeted tyrosine kinase inhibitors (SMV-TKIs) were linked to higher reporting of PAAD and PAD, whereas anti-VEGF monoclonal antibodies (anti-VMAs) were associated with elevated reporting across all outcomes. Time-to-onset analyses uncovered temporal heterogeneity: PAAD and PAA occurred early in FAERS (median 29.5 days and 25.0 days, respectively), whereas PAD followed an early- and late-onset pattern. Within JADER, PAA events presented early, while PAD showed delayed onset (median 853.0 days). Multivariable regression supported significant independent associations between AI exposure and both PAAD and PAD, with adjusted Odds Ratios of 4.078 and 7.275 in FAERS and greater effect sizes in JADER. Time-window stratification confirmed dual reporting peaks in the early (0–29-day) and long-term (≥360-day) windows.

**Conclusion:**

In conclusion, subclass-specific and time-dependent disproportional reporting signals of AI-associated PAAD were identified. Anti-VMAs were associated with elevated signals for PAAD, PAA, and PAD, whereas SMV-TKIs were mainly linked to PAAD and PAD. PAAD and PAA occurred predominantly early after drug initiation, whereas PAD showed prominent delayed onset.

## 1 Introduction

Anti-angiogenic targeted therapies have revolutionized treatment paradigms for a broad spectrum of malignancies (Ferrara and Kerbel, 2005; Folkman, 2007). However, their widespread clinical adoption has raised growing safety concerns, particularly regarding vascular adverse events. Vascular endothelial growth factor (VEGF) and vascular endothelial growth factor receptor (VEGFR) inhibitors, the cornerstone of anti-angiogenic regimens, selectively block tumor angiogenesis to deprive neoplastic lesions of oxygen and nutrients, thereby suppressing growth and metastatic dissemination (Ferrara et al., 2004). Across multiple solid tumors, including colorectal, non-small cell lung, and renal cell carcinomas, VEGF(R) inhibitors have consistently demonstrated significant improvements in survival (Hurwitz et al., 2004; Motzer et al., 2007).

Notably, angiogenesis inhibitor (AI) exhibited a distinct toxicity profile that diverges markedly from that of traditional cytotoxic chemotherapy, encompassing hypothyroidism, delayed wound healing, gastrointestinal perforation, and proteinuria (Eskens and Verweij, 2006; Tannir et al., 2022). Critically, because the VEGF pathway is integral to physiological angiogenesis, endothelial barrier homeostasis, and vascular tone regulation, vascular adverse events such as hypertension, arterial thromboembolism, and left ventricular dysfunction are frequently observed in patients receiving AIs therapy (Martins et al., 2019; Nalluri et al., 2008; Khakoo et al., 2008).

Emerging case reports and observational studies have recently suggested a potential association between VEGF pathway inhibitors and aortic diseases (Li et al., 2024; Uemura et al., 2025; Guyon et al., 2021). Yet, to the best of our knowledge, no study has specifically investigated the effects of AIs on the incidence and risk factors of peripheral artery aneurysm and dissection (PAAD). Owing to the rarity of PAAD, prior investigations have captured only a limited number of AIs-related cases, yielding insufficient statistical power to draw definitive conclusions (Singier et al., 2025).

To address this gap, we conducted a large-scale retrospective analysis using the U.S. Food and Drug Administration (FDA) Adverse Event Reporting System and Japanese Adverse Drug Event Report (JADER) databases (Sakaeda et al., 2013). This study aimed to evaluate the potential association between AIs and PAAD risk and to characterize the relationship between drug exposure duration and the onset of these peripheral arterial adverse events.

## 2 Methods

### 2.1 Study design and data sources

In this study, data were obtained from the FAERS and the JADER databases, which are national public pharmacovigilance databases maintained by the U.S. FDA and the Japanese Pharmaceuticals and Medical Devices Agency (PMDA), respectively. Raw data from FAERS were retrieved through the openFDA platform (https://open.fda.gov/), a public data integration initiative launched by the FDA, with all data available for download in compressed JavaScript Object Notation (JSON) format, covering reports submitted from January 2014 to December 2025. JADER data, maintained by the PMDA of Japan (https://www.pmda.go.jp/), were obtained from four core modules (DEMO, DRUG, REAC, and HIST), spanning January 2014 to June 2026.

This study was conducted in accordance with the STROBE (Strengthening the Reporting of Observational Studies in Epidemiology) guidelines and the RECORD (REporting of studies Conducted using Observational Routinely-collected health Data) extension (Cuschieri, 2019; Langan et al., 2018). Given that FAERS and JADER are publicly accessible, fully de-identified databases devoid of protected health information, the requirement for ethical review and informed consent was waived by the Institutional Review Board of Changhai Hospital, Shanghai, China.

### 2.2 Data extraction and processing

Individual case safety reports (ICSRs) meeting the pre-specified screening criteria were retrieved from the FAERS and JADER databases. Given the structured nature of these databases, custom data extraction algorithms were developed to systematically parse and map relevant variables from the raw datasets. The extracted variables encompassed: demographic characteristics (age, sex, weight); medication details (suspect drug name, start date, and end date of therapy); adverse event profiles (preferred terms, onset date, and clinical outcomes); report characteristics (reporter qualification, reporting country, and database receipt date); and relevant medical history and concomitant medications (hypertension, diabetes, fluoroquinolone use, and immunotherapy).

A rigorous data cleaning and standardization pipeline was implemented (Figure 1), involving harmonization of variable nomenclature across databases, verification of chronological logic for time-related variables, and exclusion of invalid records and extreme outliers. An adverse event record denotes one Medical Dictionary for Regulatory Activities (MedDRA)-coded reaction entry within the REAC table; a single case report may contain multiple such records. Where a single case report contained ≥1 PAAD-related adverse event record, it was counted as one PAAD event case report.

**FIGURE 1:**
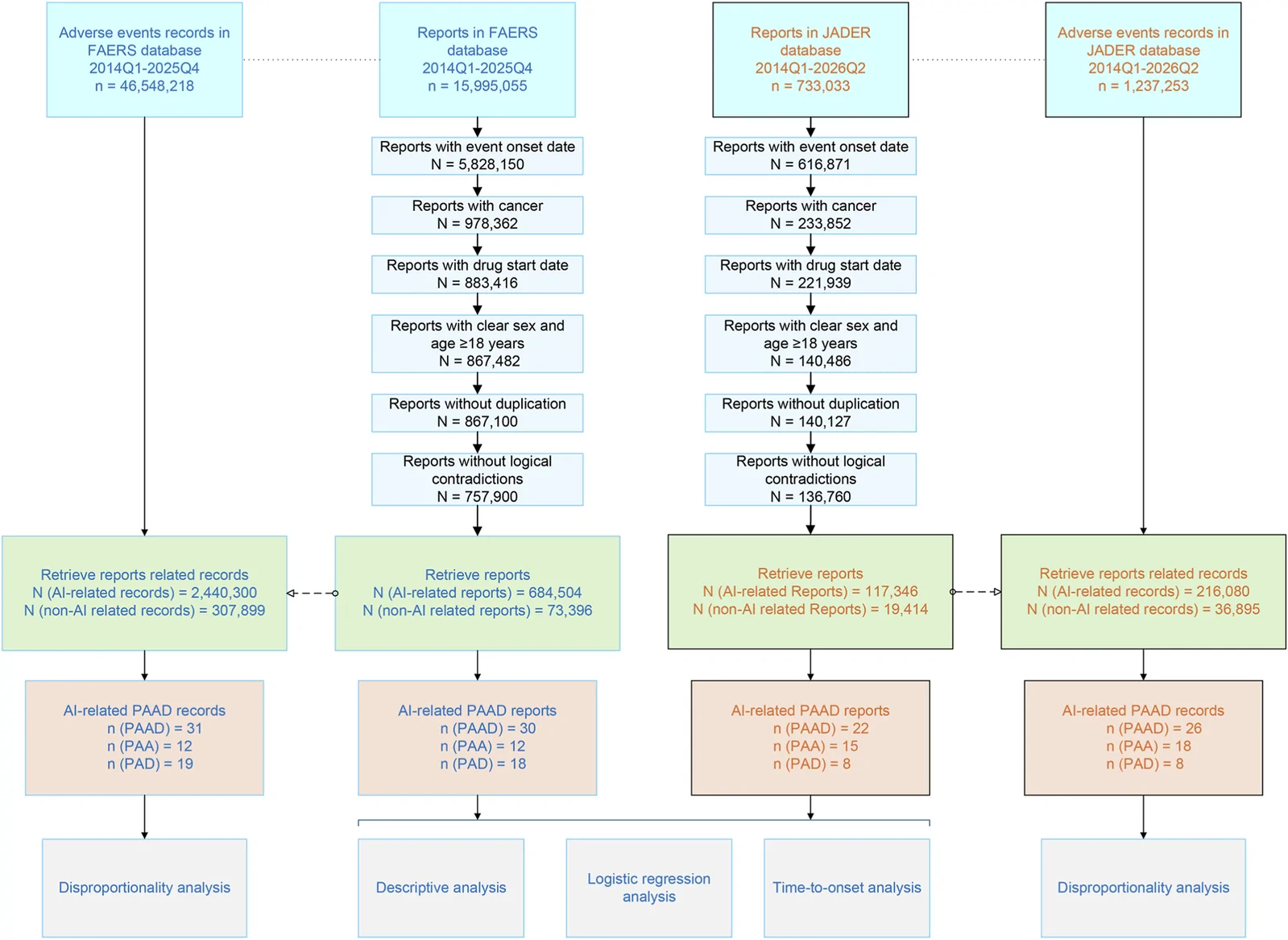
Flow chart of Reports and Adverse Events Records Selection from the FAERS and JADER databases. FAERS, U.S. Food and Drug Administration Adverse Event Reporting System; JADER, Japanese Adverse Drug Event Report Database; AI, angiogenesis inhibitor; PAAD, peripheral artery aneurysm and dissection; PAA, peripheral artery aneurysm; PAD, peripheral artery dissection.

### 2.3 Covariate definitions and missing data handling

Hypertension was identified by documented diagnosis or concurrent antihypertensive medication use. Diabetes mellitus was identified by documented diagnosis or antidiabetic medication use. Quinolone use and immunotherapy exposure were identified from corresponding medication records or MedDRA Preferred Terms (PTs).

FAERS and JADER lack dedicated fields to explicitly document the absence of comorbidities or drug exposures. Covariates for hypertension, diabetes mellitus, quinolone use, and immunotherapy exposure were ascertained using both disease-diagnosis records and corresponding medication records; a status of present (1/Yes) was assigned only when either the diagnosis or relevant medication was explicitly documented within a case report. Because covariates contained only values of 1/Yes or NA (no native true-negative entries), complete-case analysis would yield zero variance, rendering multivariable logistic regression computationally infeasible. Similarly, assuming all unreported entries represent positive status would also produce zero-variance covariates. Therefore, for the primary multivariable logistic regression, we applied a conservative assumption: unreported medical-history and exposure information was coded as absence (0/No).

### 2.4 Study population and case selection

In this study, the exposure of interest was AIs, stratified into two subclasses: small-molecule VEGFR tyrosine kinase inhibitors (SMV-TKIs; fruquintinib, cabozantinib, sunitinib, lapatinib, apatinib, surufatinib, anlotinib, lenvatinib, axitinib, sitravatinib, dovitinib, famitinib, golvatinib, derazantinib, catequentinib) and anti-VEGF monoclonal antibodies (anti-VMAs; bevacizumab, ramucirumab, aflibercept). Drugs were identified using active ingredient names or standardized codes; records involving non-target drugs due to spelling similarity or code confusion were rigorously excluded. Owing to limited event counts for individual agents, single-drug-specific analyses were not performed to avoid underpowered and unstable estimates.

Target adverse events were defined as PAAD-related events, uniformly coded per MedDRA version 27.1. PAAD comprised three categories: peripheral artery dissection (PAD), peripheral artery aneurysm (PAA), and concurrent PAD with PAA, all requiring onset dates subsequent to drug initiation. Cases involving intracranial, carotid, coronary, or pulmonary arteries, as well as pseudoaneurysms, were explicitly excluded.

The inclusion criteria were: (1) confirmed cancer diagnosis; (2) complete key fields including demographics (age, sex), medication details (drug name, start/end dates), adverse event onset date, and clinical outcome; (3) Age ≥18 years at the time of adverse event onset.

The exclusion criteria were: (1) logical contradictions (adverse event onset or drug end date preceding drug start date); (2) duplicate reports identified during the deduplication process; (3) missing exposure-start or event-onset timestamps; (4) reports judged false/invalid after manual verification by two independent researchers.

After applying the inclusion and exclusion criteria, 757,900 FAERS reports and 136,760 JADER reports with complete demographic, medication, and outcome data were retained; these reports served as the unit of analysis for both baseline characteristics and regression models. Given that a single report may document multiple adverse events, these reports corresponded to 2,748,199 and 252,975 individual adverse event records in FAERS and JADER, respectively, among which 106 and 44 were classified as PAAD events.

### 2.5 Statistical analysis methods

Disproportionality analyses were performed at the adverse event record level using raw unfiltered data. Descriptive statistics, logistic regression, and time-to-onset (TTO) analyses were conducted at the case report level. Given that FAERS and JADER lack unique patient identifiers, the true number of distinct patients cannot be ascertained. Three standardized definitions were adopted throughout: (1) case reports: individual submitted adverse event reports (fundamental analytical unit of FAERS/JADER); (2) AI exposed case reports: case reports documenting treatment with angiogenesis inhibitors; (3) PAAD positive case reports: AI exposed case reports containing at least one PAAD related adverse event.

#### 2.5.1 Disproportionality analysis

The Reporting Odds Ratio (ROR) method was employed to detect potential drug-adverse event association signals (Rothman et al., 2004). To minimize confounding by indication and differential reporting patterns, oncology-related reports involving other anticancer drugs were adopted as the comparator group, rather than all non-AI reports. The ROR and its corresponding 95% confidence interval (CI) were calculated for AIs and artery disease. The formula for calculating the ROR was as follows:

$$ROR=\frac{a/c}{b/d}$$

$$95\%CI=e^{\ln\left(ROR\right)\pm 1.96\sqrt{\frac{1}{a}+\frac{1}{b}+\frac{1}{c}+\frac{1}{d}}}$$

a: Count of reports that include both the specified drug and the target adverse drug event; b: Count of reports associated with the specified drug but reporting other adverse drug events; c: Count of reports documenting the target adverse drug event but involving other drugs; d: Count of reports involving other drugs and non-target adverse drug events.

A statistically significant potential association signal between a specific drug and a specified artery adverse event was considered to be present when a ≥1 and the lower limit of the 95% CI exceeded 1.

#### 2.5.2 Logistic regression

Univariate Firth’s Penalized Logistic regression model was used to evaluate crude associations between each potential risk factor and occurrence of PAAD-event case-reports, with crude odds ratio (cOR) and its 95% CI calculated (Greenland et al., 1999; Da Silva et al., 2025). Multivariate Firth’s Penalized Logistic regression models were constructed to adjust for potential confounding factors, yielding adjusted odds ratio (aOR) and its 95% CI.

#### 2.5.3 Time-to-event analysis

Kaplan-Meier (KM), exposure window, and Weibull TTO analyses were performed at the case-report level. Denominators consisted of AI-exposed case reports with complete timestamps for both AI initiation and adverse event onset. Time zero was the reported AI start date. Exposure duration was defined as days from drug start to the earlier of drug end date or adverse event onset. Reports lacking timestamps for either exposure initiation or adverse-event onset were excluded from the time-to-event analyses. Time-to-event was the interval (days) from AI initiation to PAAD onset. Right censoring was applied to AI-exposed reports without documented target event onset within available timestamps; this reflects report-metadata-derived censoring, not clinical loss to follow-up. Given the absence of a defined source population, findings represent reporting patterns among submitted case reports, not absolute clinical risk.

#### 2.5.4 General statistical methods

Continuous variables were expressed as mean ± SD (normally distributed) or median (IQR; skewed). Categorical variables were expressed as frequency (n) and percentage (%). Continuous variables were compared using Student’s t-test or Mann-Whitney U test. Categorical variables were compared using chi-square test (expected cell frequency ≥5) or Fisher’s exact test (Altman and Bland, 1995).

All hypothesis-tests were two-sided. For primary association analyses, the Benjamini-Hochberg false-discovery-rate (FDR-BH) procedure was applied to address multiple comparisons. Both nominal unadjusted *P*-values and FDR-corrected *q*-values are reported; statistical inference for primary outcomes relied on FDR-corrected *q*-values. All OR, 95% CI and *P*-value estimates were exported directly from fitted statistical models to eliminate rounding-driven inconsistencies between confidence intervals and *P*-values. *q*-values <0.05 were considered statistically significant. Exploratory analyses (temporal patterns, JADER-stratified models) are hypothesis-generating; over-reliance on nominal significance should be avoided.

All analyses were conducted in R (version 4.5.3, https://www.r-project.org/; R Foundation for Statistical Computing).

## 3 Results

### 3.1 Baseline characteristics in the FAERS and JADER cohorts

A total of 757,900 cases from FAERS (Table 1) and 136,760 cases from JADER (Supplementary Table S1) were included, with 73,396 and 19,414 reports involving AI exposure, respectively.

**TABLE 1:** Baseline characteristics of the cases in the FAERS cohort.

Characteristics	Non-AI	AI	*P*-value	*q*-value
FAERS cohort	N = 684,504	N = 73,396		
Age (years, median [IQR])	67.0 (58.0, 75.0)	66.0 (58.0, 73.0)	<0.001	<0.001
Age group (years)	​	​	<0.001	<0.001
<70 years	395,755 (57.8%)	45,907 (62.5%)	​	​
≥70 years	288,749 (42.2%)	27,489 (37.5%)	​	​
Gender	​	​	<0.001	<0.001
Male	339,152 (49.5%)	40,554 (55.3%)	​	​
Female	345,352 (50.5%)	32,842 (44.7%)	​	​
Weight (kg, median [IQR])	69.9 (58.9, 83.0)	67.5 (57.0, 80.7)	<0.001	<0.001
Weight group (kg)	​	​	<0.001	<0.001
<70 kg	157,015 (50.0%)	19,567 (54.5%)	​	​
≥70 kg	156,752 (50.0%)	16,342 (45.5%)	​	​
Missing	370,737 (54.2%)	37,487 (51.1%)	​	​
Hypertension	105,354 (15.4%)	16,122 (22.0%)	<0.001	<0.001
Diabetes	66,685 (9.7%)	7,158 (9.8%)	0.927	0.927
Quinolones	18,304 (2.7%)	1,132 (1.5%)	<0.001	<0.001
Immunotherapy	87,424 (12.8%)	21,818 (29.7%)	<0.001	<0.001
Reporters type	​	​	<0.001	<0.001
Health expert	512,558 (75.6%)	52,547 (71.9%)	​	​
Non-health expert	164,999 (24.4%)	20,552 (28.1%)	​	​
Missing	6,947 (1.0%)	297 (0.4%)	​	​

FAERS, U.S., food and drug administration adverse event reporting system; AI, angiogenesis inhibitor; IQR, interquartile range; *q*-value, multiple-testing adjusted *P*-value.

Within the FAERS cohort (Table 1), reports in the AI group were significantly younger than those in the non-AI group (median 66.0 [IQR: 58.0–73.0] vs. 67.0 [IQR: 58.0–75.0] years; *q*-value <0.001), with a higher proportion aged <70 years (62.5% vs. 57.8%; *q*-value <0.001). Males were more frequently represented in the AI group (55.3% vs. 49.5%; *q*-value <0.001). Regarding clinical characteristics, AI-exposed reports had a lower median body weight (67.5 kg [IQR: 57.0–80.7] vs. 69.9 kg [IQR: 58.9–83.0]; *q*-value <0.001) and a higher proportion weighing <70 kg (54.5% vs. 50.0%; *q*-value <0.001); notably, weight data were less frequently missing in the AI group (51.1% vs. 54.2%). For comorbidities and concomitant medications, the AI group had higher hypertension prevalence (22.0% vs. 15.4%; *q*-value <0.001), lower quinolone use (1.5% vs. 2.7%; *q*-value <0.001), and markedly higher immunotherapy use (29.7% vs. 12.8%; *q*-value <0.001). Diabetes prevalence was comparable between groups (9.8% vs. 9.7%; *q*-value = 0.927). Reports submitted by healthcare professionals were less common in the AI group (71.9% vs. 75.6%; *q*-value <0.001).

In the JADER cohort (Supplementary Table S1), baseline characteristics also differed across groups, albeit with patterns distinct from those observed in FAERS. AI-exposed patients had a higher proportion of individuals aged <70 years (53.0% vs. 48.0%; *q*-value <0.001), alongside slightly higher male representation (42.6% vs. 40.6%; *q*-value <0.001). Of note, completeness of weight data represented a major limitation: missing rates were substantially higher in the AI group relative to the non-AI group (52.3% vs. 39.0%). Among patients with non-missing weight records, individuals weighing ≥70 kg were more prevalent in the AI group (16.4% vs. 13.6%; *q*-value <0.001). Regarding comorbidities, AI-exposed patients showed higher hypertension prevalence (27.8% vs. 22.8%; *q*-value <0.001) and lower diabetes prevalence (10.8% vs. 12.7%; *q*-value <0.001). Concomitant medications also differed substantially between groups: quinolone use was less common among AI-exposed patients (0.9% vs. 3.1%; *q*-value <0.001), while immunotherapy use was markedly higher (39.0% vs. 29.9%; *q*-value <0.001). Lastly, nearly all reports were submitted by healthcare professionals; despite attaining statistical significance after adjustment, the absolute difference between groups was minimal (99.9% vs. 99.3%; *q*-value <0.001).

### 3.2 Disproportionality analysis of AI-Related PAAD in the FAERS and JADER cohorts

Disproportionality analyses were conducted using 2,748,199 adverse events in the FAERS cohort (Table 2). AI exposure was associated with significantly elevated RORs for the composite PAAD endpoint (ROR = 3.276; 95% CI: 2.156–4.979) and PAD (ROR = 6.275; 95% CI: 3.437–11.455), whereas the association for PAA failed to attain statistical significance (ROR = 1.865; 95% CI: 0.994–3.498). Subgroup analyses stratified by drug mechanism of action revealed distinct safety profiles across AI subtypes. Specifically, SMV-TKIs in FAERS showed significant signals for PAAD (ROR = 2.597; 95% CI: 1.553–4.344) and PAD (ROR = 5.861; 95% CI: 2.984–11.511), but not for PAA (ROR = 1.061; 95% CI: 0.423–2.658). Conversely, anti-VMAs exhibited significant disproportionate signals for all three endpoints: PAAD (ROR = 5.136; 95% CI: 2.850–9.254), PAA (ROR = 4.067; 95% CI: 1.845–8.961), and PAD (ROR = 7.407; 95% CI: 3.028–18.121).

**TABLE 2:** Disproportionality analysis of AI-Related PAAD in the FAERS cohort.

Characteristic	Overall events	PAAD events	ROR (95% CI)
FAERS cohort			
PAAD	​	​	3.276 (2.156, 4.979)
Non-AI	2,440,300	75	​
AI	307,899	31	​
PAA	​	​	1.865 (0.994, 3.498)
Non-AI	2,440,300	51	​
AI	307,899	12	​
PAD	​	​	6.275 (3.437, 11.455)
Non-AI	2,440,300	24	​
AI	307,899	19	​
SMV-TKIs subgroup			
PAAD	​	​	2.597 (1.553, 4.344)
Non-AI	2,440,300	75	​
AI	225,528	18	​
PAA	​	​	1.061 (0.423, 2.658)
Non-AI	2,440,300	51	​
AI	225,528	5	​
PAD	​	​	5.861 (2.984, 11.511)
Non-AI	2,440,300	24	​
AI	225,528	13	​
Anti-VMAs subgroup			
PAAD	​	​	5.136 (2.850, 9.254)
Non-AI	2,440,300	75	​
AI	82,371	13	​
PAA	​	​	4.067 (1.845, 8.961)
Non-AI	2,440,300	51	​
AI	82,371	7	​
PAD	​	​	7.407 (3.028, 18.121)
Non-AI	2,440,300	24	​
AI	82,371	6	​

FAERS, U.S., food and drug administration adverse event reporting system; AI, angiogenesis inhibitor; SMV-TKIs, small-molecule VEGFR, tyrosine kinase inhibitors; VEGFR, vascular endothelial growth factor receptor; Anti-VMAs, anti-VEGF, monoclonal antibodies; PAAD, peripheral artery aneurysm and dissection; PAA, peripheral artery aneurysm; PAD, peripheral artery dissection; ROR, reporting odds ratio; CI, confidence interval.

Validation in the JADER cohort yielded stronger effect estimates using 252,975 adverse events (Supplementary Table S2), with all three arterial endpoints reaching statistical significance and demonstrating markedly higher RORs than those observed in FAERS: PAAD (ROR = 8.465; 95% CI: 4.641–15.441), PAA (ROR = 8.113; 95% CI: 3.975–16.559), and PAD (ROR = 9.372; 95% CI: 3.066–28.651). For SMV-TKIs, significant associations were confirmed for PAAD (ROR = 6.533; 95% CI: 3.085–13.834), PAA (ROR = 3.288; 95% CI: 1.072–10.086), and PAD (ROR = 14.964; 95% CI: 4.749–47.154). Similarly, anti-VMAs showed corroborated elevated RORs for PAAD (ROR = 10.809; 95% CI: 5.446–21.451) and PAA (ROR = 13.968; 95% CI: 6.564–29.720), whereas the anti-VMA-PAD association was non-significant (ROR = 2.592; 95% CI: 0.303–22.189), likely due to only one eligible event (n = 1).

Drug-specific disproportionality signals and the anatomical distribution of adverse events were evaluated. In the FAERS cohort, statistically significant positive signal was identified for aflibercept-associated PAD (ROR = 42.077, 95% CI: 9.941–178.090; Supplementary Table S3), bevacizumab-associated PAD (ROR = 8.020, 95% CI: 1.010–19.620; Supplementary Table S3), lenvatinib-associated PAD (ROR = 13.991, 95% CI: 1.504–29.258; Supplementary Table S3), and ramucirumab-associated PAA (ROR = 33.390, 95% CI: 1.658–92.416; Supplementary Table S3). In the JADER cohort, consistent significant signals were detected for bevacizumab-associated PAA (ROR = 9.017, 95% CI: 1.037–22.604; Supplementary Table S3), lenvatinib-associated PAD (ROR = 25.547, 95% CI: 1.296–80.506; Supplementary Table S3), and ramucirumab-associated PAA (ROR = 31.831, 95% CI: 1.792–79.830; Supplementary Table S3). Subsequent stratification of PAAD events by anatomical distribution revealed distinct patterns across cohorts. In FAERS, peripheral artery dissection, not otherwise specified (NOS), exhibited a prominent and statistically robust signal (ROR = 12.882, 95% CI: 5.339–31.080; Supplementary Table S4), whereas peripheral artery aneurysm NOS and other regionally specified arterial events did not reach statistical significance. Conversely, most anatomically stratified analyses in the JADER cohort yielded confidence intervals crossing unity, with peripheral artery aneurysm NOS, demonstrating only borderline significance (ROR = 5.858, 95% CI: 0.999–12.638; Supplementary Table S4). Furthermore, ROR calculation was precluded for several rare anatomical phenotypes due to zero events in the non-AI comparator group.

### 3.3 Time-to-onset analysis of AI-related PAAD

In the FAERS cohort, Kaplan-Meier survival analysis and onset-time distribution analysis were performed to characterize the temporal pattern of AI-associated arterial adverse events. For all AI-exposed reports, composite PAAD (n = 30) showed an early reporting pattern (β = 0.708; 95% CI: 0.547–0.915; median TTO = 29.5 days, IQR: 15.0–311.0; Table 3), with 50.0% of events within 0–29 days and a late-onset tail (23.3% ≥ 360 days; Figures 2A,B). PAA (n = 12) had a median TTO of 25.0 days (IQR: 13.0–203.0; Table 3), with 58.3% of cases occurring within the first month (Figures 2C,D). By contrast, PAD had a broad, heterogeneous distribution (median TTO = 36.0 days, IQR: 15.0–434.0; Table 3), including early events (44.4%, 0–29 days) and a prominent late-onset tail (27.8% ≥ 360 days; Figures 2E,F). Both SMV-TKIs and anti-VMAs subgroups exhibited early-predominant reporting profiles (all β < 1.0; Table 3). The SMV-TKI subgroup showed higher proportions of early events: 52.9% for PAAD (Supplementary Figures S1A,B), 60.0% for PAA (Supplementary Figures S1C,D), and 50.0% for PAD (Supplementary Figures S1E,F) within 0–29 days. In the anti-VMA subgroup, 46.2% of PAAD (Supplementary Figures S2A,B) cases and 57.1% of PAA (Supplementary Figures S2C,D) cases occurred within 0–29 days; PAD cases were evenly distributed between early-onset (33.3%, 0–29 days) and late-onset (33.3%, ≥360 days; Supplementary Figure S2E,F).

**TABLE 3:** Time-to-onset analysis of AI-Related PAAD by Weibull distribution analysis in the FAERS cohort.

Characteristics	TTO (days)		Weibull distribution		
	Case reports	Median (d) (IQR)	Scale parameter: log(α) (95% CI)	Shape parameter: β (95% CI)	Pattern
FAERS cohort					
PAAD	30	29.5 (15.0, 311.0)	15.589 (12.921, 18.258)	0.708 (0.547, 0.915)	Early dominant
PAA	12	25.0 (13.0, 203.0)	17.594 (12.420, 22.769)	0.666 (0.440, 1.010)	Early trend
PAD	18	36.0 (15.0, 434.0)	15.881 (12.416, 19.346)	0.737 (0.531, 1.021)	Early trend
SMV-TKIs subgroup					
PAAD	17	28.0 (20.0, 311.0)	15.546 (12.024, 19.068)	0.725 (0.517, 1.018)	Early trend
PAA	5	22.0 (21.0, 255.0)	16.809 (9.677, 23.940)	0.752 (0.406, 1.394)	Early trend
PAD	12	29.5 (17.5, 372.5)	16.189 (11.705, 20.673)	0.714 (0.476, 1.073)	Early trend
Anti-VMAs subgroup					
PAAD	13	43.0 (15.0, 151.0)	15.752 (11.602, 19.901)	0.673 (0.453, 1.000)	Early dominant
PAA	7	28.0 (8.0, 151.0)	18.194 (10.831, 25.558)	0.593 (0.337, 1.042)	Early trend
PAD	6	87.0 (15.0, 462.0)	15.283 (9.891, 20.675)	0.758 (0.452, 1.364)	Early trend

FAERS, U.S., food and drug administration adverse event reporting system; AI, angiogenesis inhibitor; SMV-TKIs, small-molecule VEGFR, tyrosine kinase inhibitors; VEGFR, vascular endothelial growth factor receptor; Anti-VMAs, anti-VEGF, monoclonal antibodies; PAAD, peripheral artery aneurysm and dissection; PAA, peripheral artery aneurysm; PAD, peripheral artery dissection; TTO, time-to-onset; IQR, interquartile range; CI, confidence interval.

**FIGURE 2:**
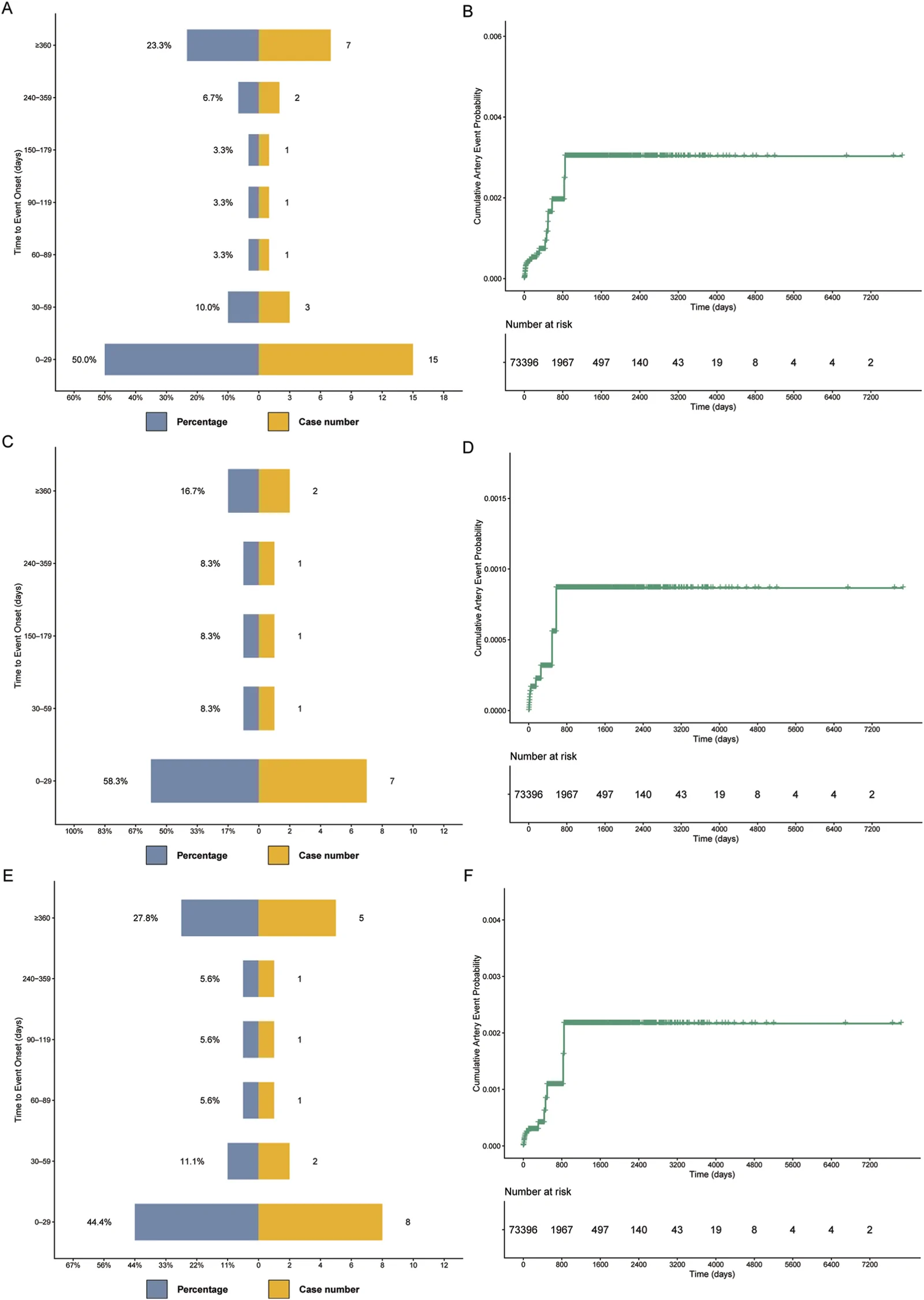
Time-to-Onset Analysis of AI-Related PAAD in the FAERS Cohort. **(A,B)** Time-to-Onset analysis of PAAD; **(C,D)** Time-to-Onset analysis of PAA; **(E,F)** Time-to-Onset analysis of PAD. FAERS, U.S. Food and Drug Administration Adverse Event Reporting System; AI, angiogenesis inhibitor; PAAD, peripheral artery aneurysm and dissection; PAA, peripheral artery aneurysm; PAD, peripheral artery dissection.

In the JADER cohort, TTO distributions exhibited distinct temporal heterogeneity across endpoints. The composite PAAD exhibited an overall early-predominant reporting pattern (n = 22, median TTO: 50.0 days, IQR: 27.0–151.0; Supplementary Table S5), primarily driven by PAA (n = 15, β = 0.622; median TTO: 43.0 days, IQR: 23.0–84.0; Supplementary Table S5) with 31.8% of overall PAAD events occurred within 0–29 days (Figures 3A,B). 40.0% of overall PAA events occurred within 0–29 days (Figures 3C,D). Conversely, PAD displayed a markedly right-skewed, late-dominant reporting distribution (n = 8, β = 1.276; median TTO: 853.0 days, IQR: 28.0–859.0; Supplementary Table S5), with 62.5% of PAD events occurring ≥360 days (Figures 3E,F). Subclass analysis confirmed this divergent temporal pattern. Notably, the composite PAAD (Figures 3A,B) endpoint for SMV-TKIs exhibited a constant-pattern distribution (β = 0.992; Supplementary Table S5) with 40.0% of PAAD events occurring within 0–29 days and 40.0% emerging at ≥360 days. Within the SMV-TKIs subgroup, 75.0% of PAA (Supplementary Figures S3C,D) events arose within the initial 29 days, while 57.1% of PAD events occurred ≥360 days alongside a small proportion of early-onset PAD cases (Supplementary Figures S3E,F). Among anti-VMA-exposed reports, 58.3% of PAAD events (Supplementary Figures S4A,B) and 63.7% of PAA events (Supplementary Figures S4C,D) occurred within 0–59 days, whereas all observed PAD events emerged at ≥360 days (Supplementary Figures S4E,F).

**FIGURE 3:**
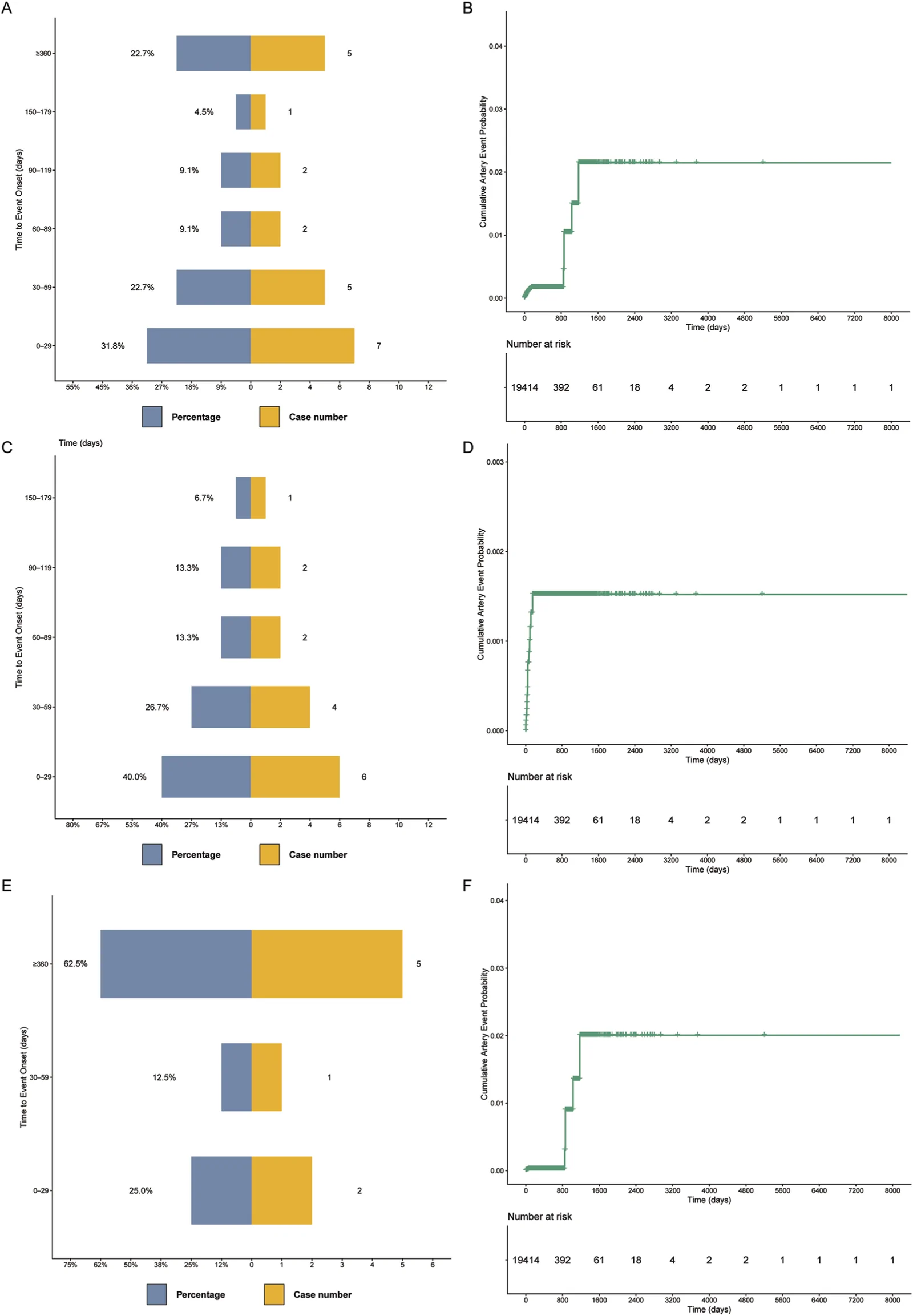
Time-to-Onset Analysis of AI-Related PAAD in the JADER Cohort. **(A,B)** Time-to-Onset analysis of PAAD; **(C,D)** Time-to-Onset analysis of PAA; **(E,F)** Time-to-Onset analysis of PAD. JADER, Japanese Adverse Drug Event Report Database; AI, angiogenesis inhibitor; PAAD, peripheral artery aneurysm and dissection; PAA, peripheral artery aneurysm; PAD, peripheral artery dissection.

### 3.4 Logistic regression analysis of factors associated with AI-related PAAD

#### 3.4.1 Analysis of factors associated with AI-related PAAD in the FAERS cohort

In univariate models (Supplementary Table S6), AI exposure showed a statistically significant association with PAAD (cOR = 3.925; 95% CI: 2.535–5.929; *q*-value <0.001) and PAD (cOR = 7.343; 95% CI: 3.945–13.468; *q*-value <0.001), but not with PAA. Subgroup analysis by AI subtype revealed distinct patterns. Anti-VMAs were significantly associated with all three endpoints: PAAD (cOR = 5.421; 95% CI: 2.902–9.367), PAA (cOR = 4.410; 95% CI: 1.880–8.932), and PAD (cOR = 8.050; 95% CI: 3.093–18.120; all *q*-values <0.05). In contrast, SMV-TKIs exhibited significant associations only with PAAD (cOR = 3.314; 95% CI: 1.905–5.450) and PAD (cOR = 7.302; 95% CI: 3.564–14.254; both *q*-values <0.001). Among predefined covariates, age ≥70 years (cOR = 0.354; 95% CI: 0.156–0.718; *q*-value = 0.008), and non-healthcare professional reporting (cOR = 0.277; 95% CI: 0.076–0.721; *q*-value = 0.012) were significantly associated with lower reporting odds of PAD. No significant associations were observed for the remaining covariates.

After multivariable adjustment in the FAERS cohort, AI exposure remained significantly associated with PAAD (aOR = 4.078; 95% CI: 2.602–6.241; *q*-value <0.001; Figure 4A) and PAD (aOR = 7.275; 95% CI: 3.825–13.618; *q*-value <0.001; Figure 4B), but not for PAA (aOR = 2.555; 95% CI: 1.299–4.664; *q*-value = 0.065; Figure 4C). Subgroup analyses stratified by AI subtype further elucidated distinct safety profiles in FAERS. Anti-VMAs remained significantly associated with all three arterial endpoints: PAAD (aOR = 5.090; 95% CI: 2.712–8.853; *q*-value <0.001; Supplementary Table S7), PAA (aOR = 4.709; 95% CI: 1.993–9.644; *q*-value = 0.010; Supplementary Table S7), and PAD (aOR = 6.504; 95% CI: 2.489–14.722; *q*-value = 0.002; Supplementary Table S7). SMV-TKIs showed significant associations with PAAD (aOR = 3.593; 95% CI: 2.032–6.026; *q*-value <0.001; Supplementary Table S7) and PAD (aOR = 8.180; 95% CI: 3.854–16.573; *q*-value <0.001; Supplementary Table S7), but not with PAA (aOR = 1.639; 95% CI: 0.593–3.694; *q*-value = 0.558; Supplementary Table S7). Advanced age (≥70 years) retained the association specifically with a lower aOR for PAD (aOR = 0.401; 95% CI: 0.176–0.816; *q*-value = 0.028; Figure 4B). Reports submitted by non-healthcare professionals were associated with lower odds of PAD (aOR = 0.259; 95% CI: 0.070–0.678; *q*-value = 0.015; Figure 4B). All other covariates showed no independent associations after adjustment (Figures 4A–C).

**FIGURE 4:**
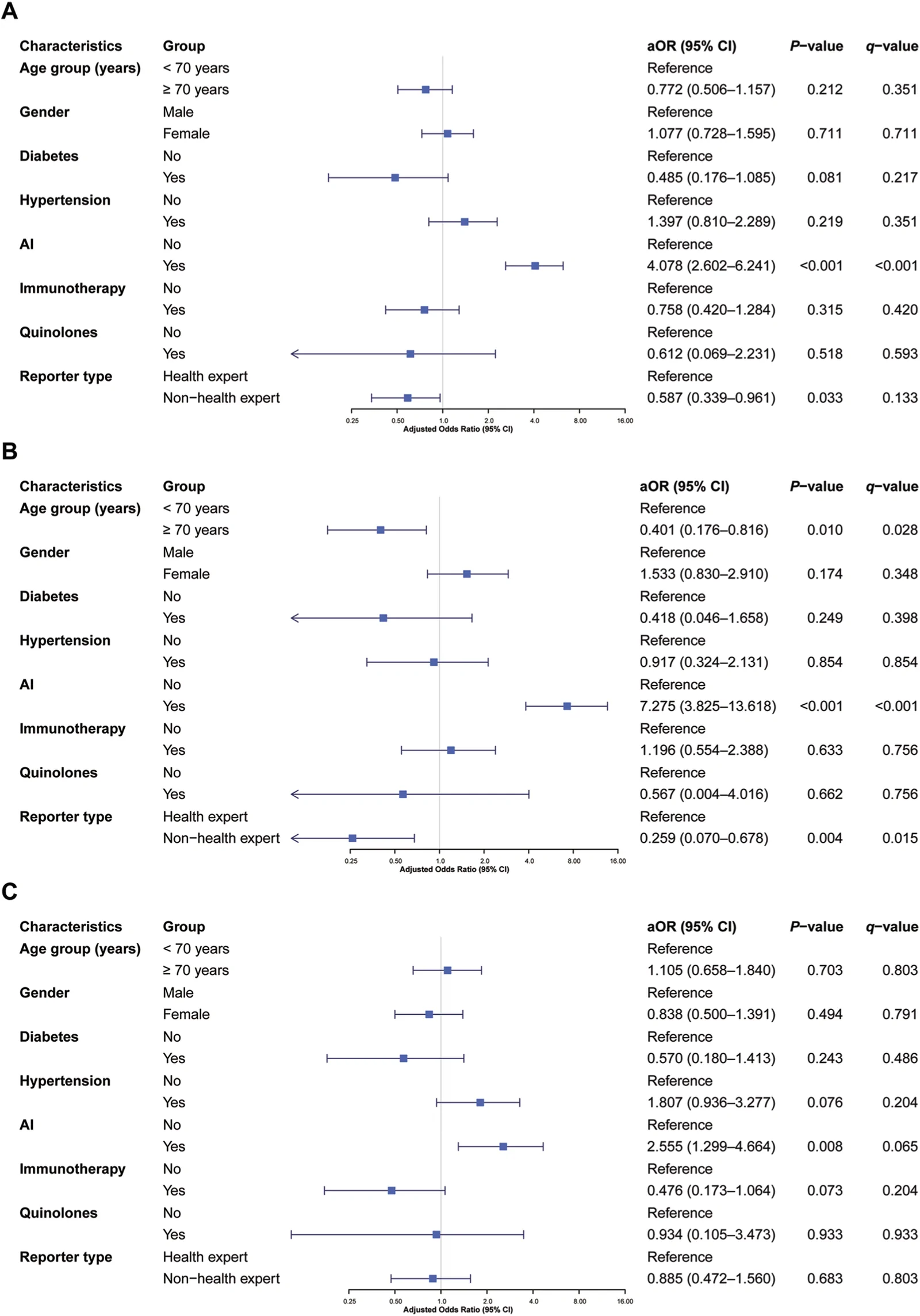
Multivariate Logistic Regression Analysis of Factors Associated with AI-Related PAAD in the FAERS Cohort. **(A)** Analysis of PAAD; **(B)** Analysis of PAD; **(C)** Analysis of PAA. FAERS, U.S. Food and Drug Administration Adverse Event Reporting System; AI, angiogenesis inhibitor; PAAD, peripheral artery aneurysm and dissection; PAA, peripheral artery aneurysm; PAD, peripheral artery dissection; aOR, adjusted odds ratio; CI, confidence interval; *q*-value, multiple-testing adjusted *P*-value.

#### 3.4.2 Analysis of factors associated with AI-related PAAD in the JADER cohort

Validation in the JADER cohort generally yielded larger point estimates. In univariate models (Supplementary Table S8), AI exposure showed the strong association across all endpoints in the JADER cohort, with notable cORs for PAD (cOR = 11.421; 95% CI: 3.779–39.630; *q*-value <0.001), PAAD (cOR = 8.250; 95% CI: 4.392–15.824; *q*-value <0.001), and PAA (cOR = 7.500; 95% CI: 3.564–16.101; *q*-value <0.001). Subgroup analyses revealed subtype-specific association patterns. SMV-TKIs were significantly associated with PAAD (cOR = 7.497; 95% CI: 3.347–16.052; *q*-value <0.001) and PAD (cOR = 19.631; 95% CI: 6.251–69.336; *q*-value <0.001), but not with PAA (cOR = 4.239; 95% CI: 1.277–11.664; *q*-value = 0.069). Anti-VMAs yielded significant associations with PAAD (cOR = 9.422; 95% CI: 4.424–19.572; *q*-value <0.001) and PAA (cOR = 11.442; 95% CI: 5.058–25.652; *q*-value <0.001). Furthermore, age ≥70 years was significantly associated with lower reporting odds of PAAD (cOR = 0.306; 95% CI: 0.139–0.613; *q*-value = 0.002) and PAD (cOR = 0.124; 95% CI: 0.013–0.519; *q*-value = 0.009), but not with PAA (cOR = 0.414; 95% CI: 0.175–0.899; *q*-value = 0.069). No other covariates exhibited significant independent associations.

Multivariable logistic regression confirmed AI exposure as the factor strongly associated with PAAD (aOR = 7.853; 95% CI: 4.133–15.249; *q*-value <0.001; Figure 5A), PAD (aOR = 9.366; 95% CI: 3.021–33.061; *q*-value <0.001; Figure 5B), and PAA (aOR = 7.535; 95% CI: 3.533–16.434; *q*-value <0.001; Figure 5C). Regarding AI subtypes, SMV-TKIs were significantly associated with PAAD (aOR = 7.641; 95% CI: 3.346–16.734; *q*-value <0.001; Supplementary Table S9) and PAD (aOR = 18.075; 95% CI: 5.298–68.336; *q*-value <0.001; Supplementary Table S9), but not with PAA (aOR = 4.612; 95% CI: 1.373–12.928; *q*-value = 0.059; Supplementary Table S9). Anti-VMAs exhibited significant associations with PAAD (aOR = 8.352; 95% CI: 3.887–17.537; *q*-value <0.001; Supplementary Table S9) and PAA (aOR = 10.562; 95% CI: 4.606–24.094; *q*-value <0.001; Supplementary Table S9). The association between Anti-VMAs and PAD failed to reach statistical significance (aOR = 3.396; 95% CI: 0.339–18.530; *q*-value = 0.414; Supplementary Table S9). Advanced age (≥70 years) was significantly associated with lower reporting odds for PAAD (aOR = 0.326; 95% CI: 0.147–0.658; *q*-value = 0.006; Figure 5A) and PAD (aOR = 0.150; 95% CI: 0.016–0.638; *q*-value = 0.033; Figure 5B), but not with PAA (aOR = 0.420; 95% CI: 0.176–0.921; *q*-value = 0.067; Figure 5C). No other covariates showed significant associations (Figures 5A–C).

**FIGURE 5:**
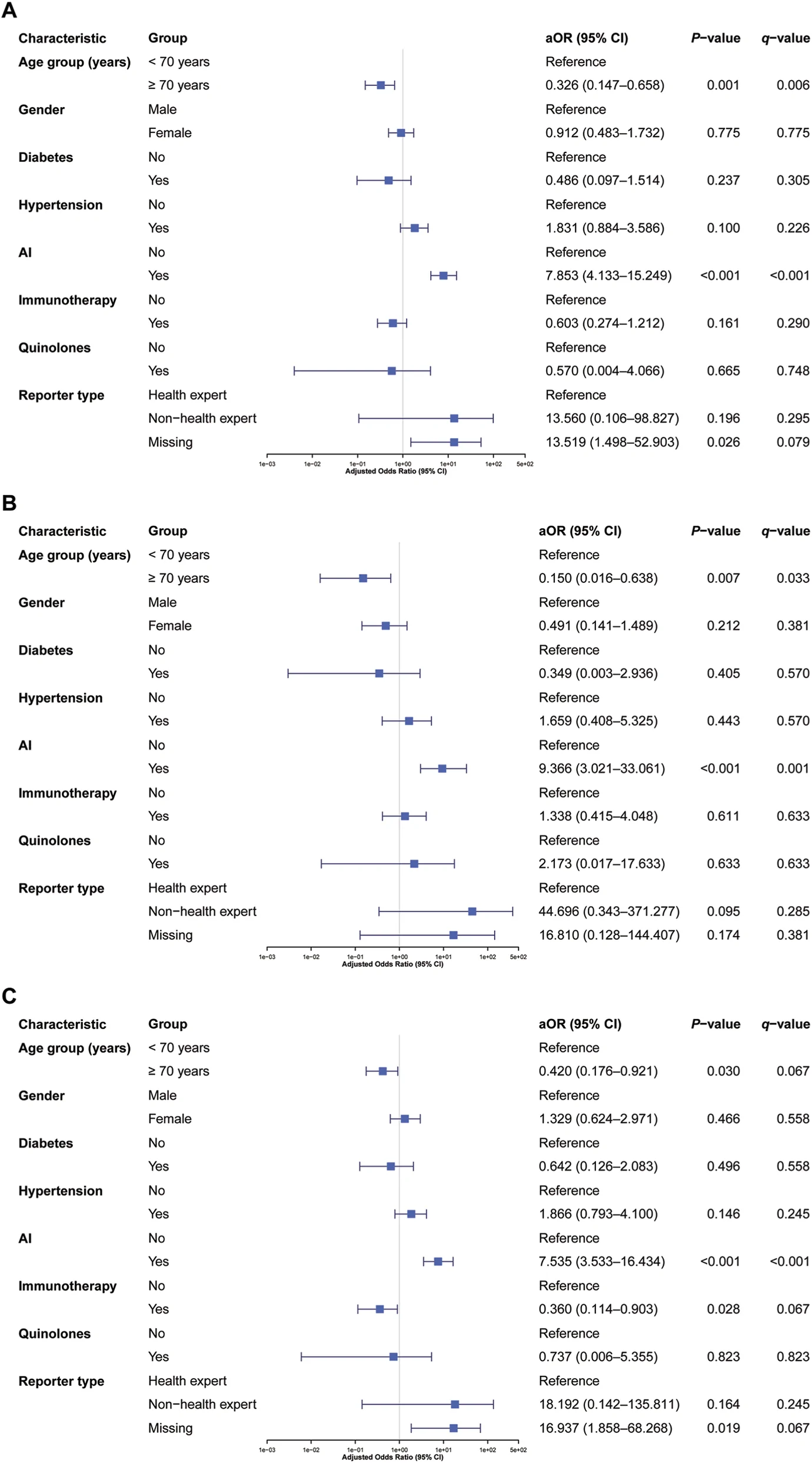
Multivariate Logistic Regression Analysis of Factors Associated with AI-Related PAAD in the JADER Cohort. **(A)** Analysis of PAAD; **(B)** Analysis of PAD; **(C)** Analysis of PAA. JADER, Japanese Adverse Drug Event Report Database; AI, angiogenesis inhibitor; PAAD, peripheral artery aneurysm and dissection; PAA, peripheral artery aneurysm; PAD, peripheral artery dissection; aOR, adjusted odds ratio; CI, confidence interval; *q*-value, multiple-testing adjusted *P*-value.

### 3.5 Temporal analysis of AI-related PAAD in the FAERS cohort

In univariate models (Table 4), early (0–29 days) and late (≥360 days) exposure windows showed statistically significant cORs for PAAD and PAD. The highest cOR values were observed at ≥360 days for PAAD (cOR = 11.572; 95% CI: 4.993–22.970; *q*-value <0.001) and PAD (cOR = 26.174; 95% CI: 9.265–61.651; *q*-value <0.001). The 30–89 days window showed a statistically significant cOR only for PAD (cOR = 6.822; 95% CI: 1.825–18.510; *q*-value = 0.013). Stratified analyses according to drug subclass revealed subclass-specific patterns (Table 4). For SMV-TKIs, statistically significant cORs were observed for PAAD across 0–29 days (cOR = 3.774; 95% CI: 1.794–7.050; *q*-value = 0.003), 240–359 days (cOR = 7.019; 95% CI: 1.458–20.259; *q*-value = 0.040), and ≥360 days (cOR = 10.732; 95% CI: 3.531–24.846; *q*-value = 0.002); and for PAD across 0–29 days (cOR = 7.965; 95% CI: 3.061–17.929; *q*-value <0.001), 30–89 days (cOR = 6.789; 95% CI: 1.373–20.875; *q*-value = 0.046), 240–359 days (cOR = 12.990; 95% CI: 1.448–49.983; *q*-value = 0.046), and ≥360 days (cOR = 25.747; 95% CI: 6.887–69.880; *q*-value <0.001). No exposure time window produced a statistically significant cOR for PAA. By contrast, anti-VMAs showed statistically significant cORs for PAAD and PAD across more time windows. Specifically, statistically significant cORs for PAAD were found at 0–29 days (cOR = 5.397; 95% CI: 2.179–11.144; *q*-value = 0.003), 30–89 days (cOR = 5.597; 95% CI: 1.163–16.153; *q*-value = 0.050), 90–239 days (cOR = 5.725; 95% CI: 1.189–16.522; *q*-value = 0.050), and ≥360 days (cOR = 15.293; 95% CI: 4.232–38.693; *q*-value = 0.002); for PAD at 0–29 days (cOR = 6.402; 95% CI: 1.295–19.684; *q*-value = 0.046), 30–89 days (cOR = 10.359; 95% CI: 1.155–39.858; *q*-value = 0.050), 90–239 days (cOR = 10.595; 95% CI: 1.181–40.767; *q*-value = 0.050), and ≥360 days (cOR = 33.688; 95% CI: 6.810–103.660; *q*-value = 0.001). Among anti-VMAs, only the 0–29-day window yielded a marginally significant cOR for PAA (cOR = 5.471; 95% CI: 1.784–12.883; *q*-value = 0.056).

**TABLE 4:** Univariate temporal logistic regression analysis of AI-Associated PAAD in the FAERS cohort.

Characteristics	PAAD			PAA			PAD		
	cOR (95% CI)	P-value	*q*-value	cOR (95% CI)	P-value	*q*-value	cOR (95% CI)	P-value	*q*-value
Exposure windows (days)									
FAERS cohort nonexposure	Reference	​	​	Reference	​	​	Reference	​	​
0–29	4.165 (2.320, 7.004)	<0.001	<0.001	2.951 (1.258, 5.977)	0.016	0.078	7.045 (3.029, 14.863)	<0.001	<0.001
30–89	2.843 (0.936, 6.579)	0.063	0.079	1.388 (0.157, 5.116)	0.707	0.707	6.822 (1.825, 18.510)	0.008	0.013
90–239	1.940 (0.403, 5.596)	0.351	0.351	1.705 (0.193, 6.284)	0.553	0.691	3.591 (0.400, 13.812)	0.203	0.203
240–359	4.715 (0.980, 13.606)	0.052	0.079	4.143 (0.469, 15.275)	0.161	0.269	8.727 (0.973, 33.574)	0.052	0.065
≥360	11.572 (4.993, 22.970)	<0.001	<0.001	5.645 (1.166, 16.523)	0.035	0.087	26.174 (9.265, 61.651)	<0.001	<0.001
Subgroups nonexposure	Reference	​	​	Reference	​	​	Reference	​	​
SMV-TKIs									
0–29	3.774 (1.794, 7.050)	0.001	0.003	2.036 (0.559, 5.228)	0.245	0.350	7.965 (3.061, 17.929)	<0.001	<0.001
30–89	2.200 (0.457, 6.348)	0.275	0.344	0.644 (0.005, 4.480)	0.739	0.821	6.789 (1.373, 20.875)	0.024	0.046
90–239	0.587 (0.005, 4.042)	0.681	0.681	0.859 (0.007, 5.975)	0.913	0.913	1.810 (0.014, 12.999)	0.705	0.705
240–359	7.019 (1.458, 20.259)	0.020	0.040	6.167 (0.698, 22.741)	0.088	0.207	12.990 (1.448, 49.983)	0.028	0.046
≥360	10.732 (3.531, 24.846)	<0.001	0.002	5.236 (0.592, 19.306)	0.113	0.207	25.747 (6.887, 69.880)	<0.001	<0.001
Anti-VMAs									
0–29	5.397 (2.179, 11.144)	<0.001	0.003	5.471 (1.784, 12.883)	0.006	0.056	6.402 (1.295, 19.684)	0.027	0.046
30–89	5.597 (1.163, 16.153)	0.035	0.050	4.918 (0.556, 18.134)	0.124	0.207	10.359 (1.155, 39.858)	0.040	0.050
90–239	5.725 (1.189, 16.522)	0.033	0.050	5.030 (0.569, 18.548)	0.120	0.207	10.595 (1.181, 40.767)	0.038	0.050
240–359	2.872 (0.023, 19.796)	0.526	0.585	4.207 (0.033, 29.264)	0.413	0.516	8.861 (0.070, 63.669)	0.257	0.285
≥360	15.293 (4.232, 38.693)	<0.001	0.002	9.591 (1.085, 35.381)	0.044	0.207	33.688 (6.810, 103.660)	<0.001	0.001

FAERS, U.S., food and drug administration adverse event reporting system; AI, angiogenesis inhibitor; cOR, crude odds ratio; CI, confidence interval; *q*-value, multiple-testing adjusted *P*-value; SMV-TKIs, small-molecule VEGFR, tyrosine kinase inhibitors; VEGFR, vascular endothelial growth factor receptor; Anti-VMAs, anti-VEGF, monoclonal antibodies; PAAD, peripheral artery aneurysm and dissection; PAA, peripheral artery aneurysm; PAD, peripheral artery dissection.

After multivariable adjustment (Supplementary Table S10), significantly higher aORs for PAAD were observed at 0–29 days (aOR = 4.372; 95% CI: 2.415–7.425; *q*-value <0.001; Figure 6) and ≥360 days (aOR = 11.654; 95% CI: 4.998–23.341; *q*-value <0.001; Figure 6). No significant association was detected for PAA in any time window (all *q*-values >0.05; Figure 6). For PAD, significantly higher aORs were present at 0–29 days (aOR = 6.940; 95% CI: 2.942–14.890; *q*-value <0.001; Figure 6), 30–89 days (aOR = 6.901; 95% CI: 1.827–19.038; *q*-value = 0.023; Figure 6), and ≥360 days (aOR = 26.271; 95% CI: 9.199–62.769; *q*-value <0.001; Figure 6). Stratified multivariable regression showed that SMV-TKIs remained significantly associated with PAAD at 0–29 days (aOR = 4.158; 95% CI: 1.956–7.888; *q*-value = 0.003; Supplementary Table S11) and ≥360 days (aOR = 11.275; 95% CI: 3.676–26.508; *q*-value = 0.003; Supplementary Table S11), and with PAD at 0–29 days (aOR = 8.933; 95% CI: 3.351–20.745; *q*-value <0.001; Supplementary Table S11) and ≥360 days (aOR = 29.289; 95% CI: 7.680–82.066; *q*-value <0.001; Supplementary Table S11). No significant associations were detected for PAA in any time window (all *q*-values >0.05; Supplementary Table S11). For anti-VMAs, statistically significant associations were observed for PAAD at 0–29 days (aOR = 5.074; 95% CI: 2.042–10.532; *q*-value = 0.006; Supplementary Table S11) and ≥360 days (aOR = 14.101; 95% CI: 3.895–35.791; *q*-value = 0.003; Supplementary Table S11), and for PAD only at ≥360 days (aOR = 28.098; 95% CI: 5.670–86.759; *q*-value = 0.004; Supplementary Table S11). No other time windows reached statistical significance for anti-VMAs (all *q*-values >0.05; Supplementary Table S11).

**FIGURE 6:**
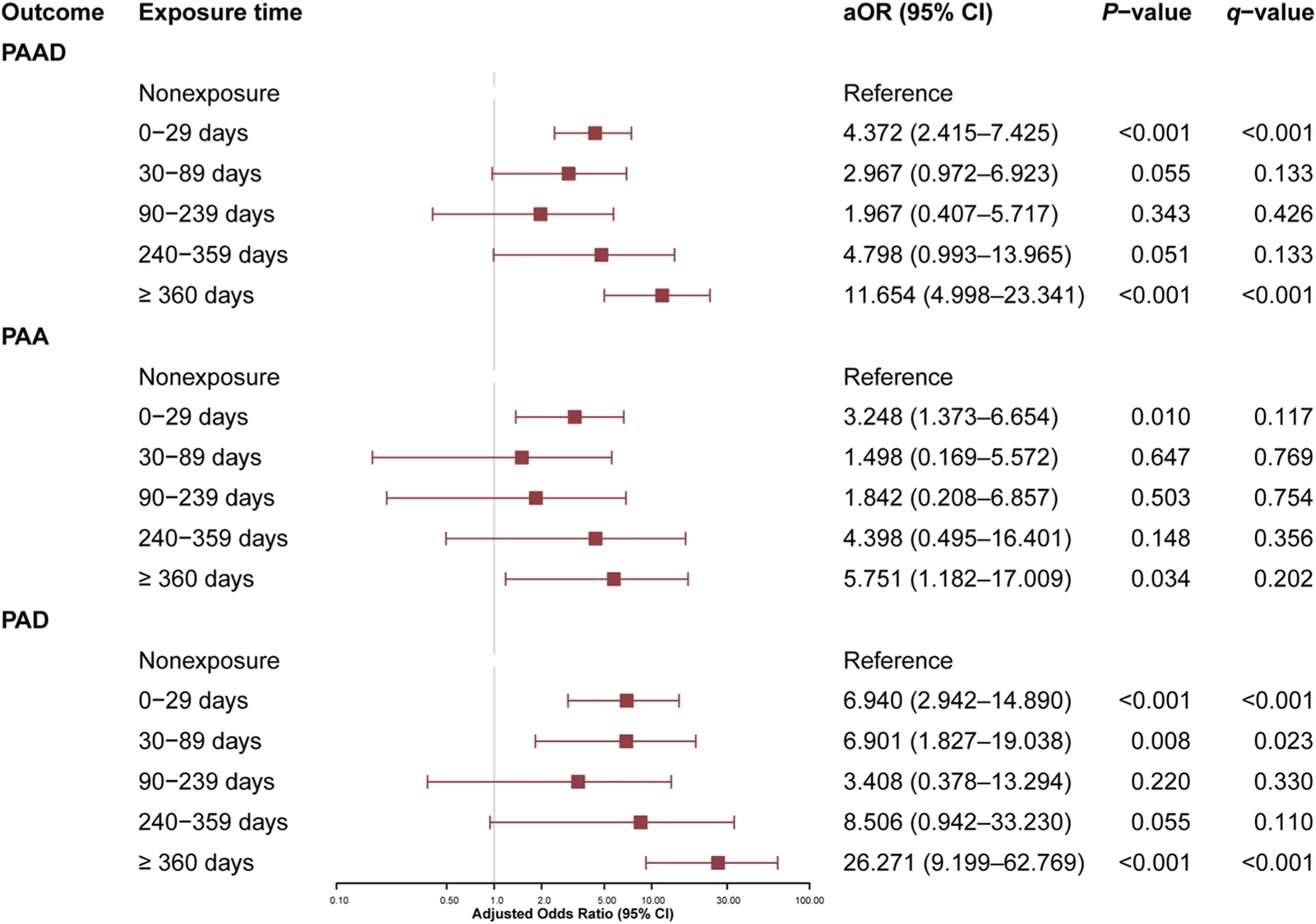
Multivariate Temporal Logistic Regression Analysis of AI-Associated PAAD in the FAERS Cohort. FAERS, U.S. Food and Drug Administration Adverse Event Reporting System; AI, angiogenesis inhibitor; PAD, peripheral artery dissection; aOR, adjusted odds ratio; CI, confidence interval; *q*-value, multiple-testing adjusted *P*-value.

### 3.6 Temporal analysis of AI-related PAAD in the JADER cohort

In univariate analyses for the JADER cohort (Table 5), early exposure windows yielded significant cORs across endpoints: 0–29-days for PAAD (cOR = 8.597; 95% CI: 3.717–18.763) and PAA (cOR = 10.153; 95% CI: 4.086–24.020; both *q*-values <0.001); 30–89-days for PAAD (cOR = 10.881; 95% CI: 4.314–24.961) and PAA (cOR = 12.445; 95% CI: 4.511–31.178; both *q*-values <0.001). The magnitude of cOR peaked in the ≥360-day window for both PAAD (cOR = 25.841; 95% CI: 8.901–64.041) and PAD (cOR = 94.760; 95% CI: 26.859–352.734), with *q*-values <0.001 for both outcomes. Stratified univariate analysis by drug subclass showed that SMV-TKIs yielded significantly higher cORs at 0–29 days for all endpoints (PAAD: cOR = 8.334, 95% CI: 2.573–21.834; PAA: cOR = 8.555, 95% CI: 2.195–25.402; PAD: cOR = 16.970, 95% CI: 2.958–76.526; all *q*-values <0.05). At 30–89 days, significance was retained only for PAAD (cOR = 6.348; 95% CI: 1.262–20.317; *q*-value = 0.048). At ≥360 days, strong significance persisted for PAAD (cOR = 44.228; 95% CI: 13.634–116.148; *q*-value <0.001) and PAD (cOR = 162.187; 95% CI: 41.818–629.027; *q*-value <0.001), while PAA remained non-significant (*q*-value = 0.371). No significant cOR was found for the 90–239 days or 240–359 days windows (all *q*-values >0.05). By comparison, anti-VMAs exhibited wider temporal response patterns: significantly higher cORs were obtained for PAAD and PAA at 0–29 days (PAAD: cOR = 9.733, 95% CI: 3.354–24.098; PAA: cOR = 12.847, 95% CI: 4.310–33.515; both *q*-values <0.001) and at 30–89 days (PAAD: cOR = 18.611, 95% CI: 6.412–46.104, *q*-value <0.001; PAA: cOR = 24.567, 95% CI: 8.238–64.117, *q*-value <0.001). At ≥360 days, significant associations were observed for PAAD (cOR = 13.493; 95% CI: 1.486–53.817; *q*-value = 0.048) and PAD (cOR = 49.480; 95% CI: 4.958–267.448; *q*-value = 0.013). No other time windows showed statistical significance for anti-VMAs (all *q*-values >0.05; Table 5).

**TABLE 5:** Univariate temporal logistic regression analysis of AI-Associated PAAD in the JADER cohort.

Characteristics	PAAD			PAA			PAD		
	cOR (95% CI)	P-value	*q*-value	cOR (95% CI)	P-value	*q*-value	cOR (95% CI)	P-value	*q*-value
Exposure windows (days)									
JADER cohort nonexposure	Reference	​	​	Reference	​	​	Reference	​	​
0–29	8.597 (3.717, 18.763)	<0.001	<0.001	10.153 (4.086, 24.020)	<0.001	<0.001	8.289 (1.445, 37.374)	0.021	0.053
30–89	10.881 (4.314, 24.961)	<0.001	<0.001	12.445 (4.511, 31.178)	<0.001	<0.001	7.970 (0.799, 43.027)	0.071	0.118
90–239	2.629 (0.290, 10.470)	0.319	0.399	3.471 (0.378, 14.338)	0.219	0.365	3.213 (0.024, 30.099)	0.501	0.501
240–359	3.337 (0.026, 24.624)	0.481	0.481	4.405 (0.034, 33.438)	0.404	0.504	12.237 (0.092, 114.702)	0.214	0.268
≥360	25.841 (8.901, 64.041)	<0.001	<0.001	3.091 (0.024, 23.454)	0.506	0.506	94.760 (26.859, 352.734)	<0.001	<0.001
Subgroup nonexposure	Reference	​	​	Reference	​	​	Reference	​	​
SMV-TKIs									
0–29	8.334 (2.573, 21.834)	0.001	0.003	8.555 (2.195, 25.402)	0.004	0.014	16.970 (2.958, 76.526)	0.004	0.013
30–89	6.348 (1.262, 20.317)	0.029	0.048	5.026 (0.547, 20.765)	0.127	0.254	13.962 (1.400, 75.385)	0.029	0.074
90–239	1.675 (0.013, 12.354)	0.740	0.740	2.211 (0.017, 16.776)	0.624	0.624	6.143 (0.046, 57.553)	0.332	0.369
240–359	7.477 (0.059, 55.262)	0.287	0.359	9.870 (0.077, 75.037)	0.241	0.371	27.420 (0.207, 257.331)	0.130	0.247
≥360	44.228 (13.634, 116.148)	<0.001	<0.001	6.451 (0.050, 49.000)	0.316	0.371	162.187 (41.818, 629.027)	<0.001	<0.001
Anti-VMAs									
0–29	9.733 (3.354, 24.098)	<0.001	<0.001	12.847 (4.310, 33.515)	<0.001	<0.001	3.240 (0.024, 30.354)	0.498	0.498
30–89	18.611 (6.412, 46.104)	<0.001	<0.001	24.567 (8.238, 64.117)	<0.001	<0.001	6.189 (0.047, 57.990)	0.330	0.369
90–239	5.514 (0.608, 21.964)	0.108	0.155	7.278 (0.793, 30.079)	0.072	0.180	6.736 (0.051, 63.116)	0.313	0.369
240–359	6.021 (0.047, 44.476)	0.330	0.366	7.948 (0.062, 60.392)	0.277	0.371	22.080 (0.167, 207.129)	0.148	0.247
≥360	13.493 (1.486, 53.817)	0.027	0.048	5.930 (0.046, 45.031)	0.334	0.371	49.480 (4.958, 267.448)	0.004	0.013

JADER, japanese adverse drug event report database; AI, angiogenesis inhibitor; cOR, crude odds ratio; CI, confidence interval; *q*-value, multiple-testing adjusted *P*-value; SMV-TKIs, small-molecule VEGFR, tyrosine kinase inhibitors; VEGFR, vascular endothelial growth factor receptor; Anti-VMAs, anti-VEGF, monoclonal antibodies; PAAD, peripheral artery aneurysm and dissection; PAA, peripheral artery aneurysm; PAD, peripheral artery dissection.

Following adjustment (Supplementary Table S12), significant association persisted at 0–29 days for PAAD (aOR = 8.319; 95% CI: 3.570–18.341; *q*-value <0.001; Figure 7) and PAA (aOR = 10.261; 95% CI: 4.085–24.662; *q*-value <0.001; Figure 7), but not for PAD (aOR = 6.954; 95% CI: 1.197–31.779; *q*-value = 0.144; Figure 7). The 30–89 days window remained significant for PAAD (aOR = 10.626; 95% CI: 4.174–24.692; *q*-value <0.001; Figure 7) and PAA (aOR = 13.010; 95% CI: 4.662–33.133; *q*-value <0.001; Figure 7). The ≥360 days window retained strong significance for PAAD (aOR = 22.649; 95% CI: 7.741–56.769; *q*-value <0.001; Figure 7) and PAD (aOR = 76.774; 95% CI: 21.403–290.295; *q*-value <0.001; Figure 7), but not for PAA (aOR = 2.790; 95% CI: 0.022–21.385; *q*-value = 0.585; Figure 7). No significant associations were found for the 90–239 days or 240–359 days windows (all *q*-values >0.05; Figure 7). Stratified multivariable regression confirmed SMV-TKIs were significant at 0–29 days for all endpoints (PAAD: aOR = 8.500, 95% CI: 2.597–22.635, *q*-value = 0.006; PAA: aOR = 9.277, 95% CI: 2.355–28.099, *q*-value = 0.021; PAD: aOR = 15.120, 95% CI: 2.538–71.431, *q*-value = 0.033; Supplementary Table S13). At ≥360 days, strong significance persisted for PAAD (aOR = 40.525; 95% CI: 12.360–108.199; *q*-value <0.001; Supplementary Table S13) and PAD (aOR = 140.426; 95% CI: 34.847–566.163; *q*-value <0.001; Supplementary Table S13), while PAA remained non-significant (aOR = 5.915; 95% CI: 0.046–45.613; *q*-value = 0.463; Supplementary Table S13). No significant associations were found for SMV-TKIs in the 30–89 days, 90–239 days, or 240–359 days windows (all *q*-values >0.05). Anti-VMAs remained significant for PAAD and PAA at 0–29 days (PAAD: aOR = 8.906, 95% CI: 3.056–22.207, *q*-value = 0.002; PAA: aOR = 12.172, 95% CI: 4.048–32.188, *q*-value <0.001; Supplementary Table S13) and at 30–89 days (PAAD: aOR = 16.690, 95% CI: 5.716–41.737, *q*-value <0.001; PAA: aOR = 22.655, 95% CI: 7.517–60.150, *q*-value <0.001; Supplementary Table S13). At ≥360 days, significance was observed only for PAD (aOR = 38.860; 95% CI: 3.845–215.025; *q*-value = 0.033; Supplementary Table S13), but not for PAAD (aOR = 11.340; 95% CI: 1.243–45.792; *q*-value = 0.080; Supplementary Table S13) or PAA (aOR = 5.282; 95% CI: 0.041–40.749; *q*-value = 0.463; Supplementary Table S13). No signal emerged for PAD in any other window, consistent with the broader temporal risk profile observed in univariate analysis (Supplementary Table S13).

**FIGURE 7:**
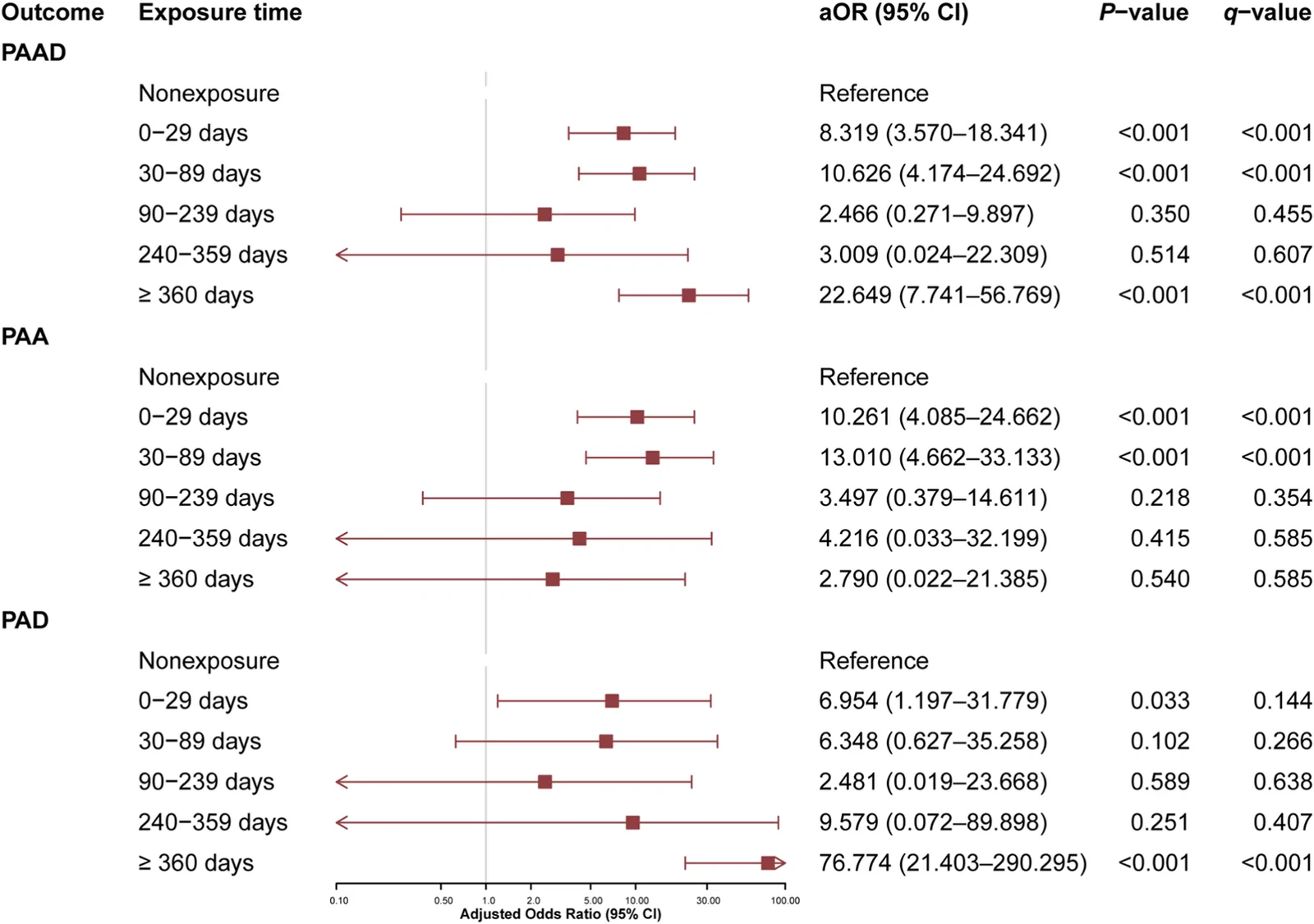
Multivariate Temporal Logistic Regression Analysis of AI-Associated PAAD in the JADER Cohort. JADER, Japanese Adverse Drug Event Report Database; AI, angiogenesis inhibitor; PAD, peripheral artery dissection; aOR, adjusted odds ratio; CI, confidence interval; *q*-value, multiple-testing adjusted *P*-.value.

## 4 Discussion

This cross-database pharmacovigilance study identified a consistent and robust disproportionate reporting signal linking AI exposure to PAAD across both FAERS and JADER. The signal was strongest for PAD, followed by the composite PAAD endpoint and PAA. Validation in the independent JADER cohort not only corroborated these associations but demonstrated even greater magnitude, particularly for PAD. These findings align with the established vascular toxicity profile of VEGF signaling pathway inhibition and corroborate prior evidence from VigiBase regarding artery dissection and aneurysm signals among individual antiangiogenic agents (Guyon et al., 2021). Importantly, while previous disproportionality studies mainly focused on the aorta (Uemura et al., 2025; Wu et al., 2024), our analysis extended the signal to the peripheral arterial tree, where PAD rather than PAA drove the majority of the composite signal. Drug-specific analyses revealed heterogeneous signal profiles across individual agents. In FAERS, significant ROR signals were detected for aflibercept, bevacizumab, and Lenvatinib associated PAD, as well as ramucirumab-associated PAA. JADER yielded consistent findings: significant signals for bevacizumab-related PAA (ROR = 9.02), lenvatinib-related PAD, and ramucirumab-related PAA. Anatomical stratification demonstrated that only peripheral artery dissection NOS exhibited the significant signal in FAERS, whereas regionally specified events and JADER anatomical subgroups showed wider confidence intervals, reflecting the inherent limitations of spontaneous reporting data in capturing precise vascular territories.

The stronger effect sizes observed in JADER warranted careful interpretation. Cross-database comparisons of spontaneous reporting system (SRS) data were inherently constrained by differences in reporting regulations, reporter composition, and healthcare-system structures. FAERS incorporated both expedited and periodic reports, whereas JADER was enriched for serious adverse events submitted predominantly by healthcare professionals. Such structural disparities could amplify signal intensity in JADER and did not, by themselves, indicate a genuinely higher biological risk in Japanese reports. Nevertheless, the fact that PAAD signals reached statistical significance across two geographically and culturally distinct databases strengthened the plausibility of a true drug-event association.

A salient finding of our study was the divergent safety profiles between anti-VMAs and SMV-TKIs. Previous analyses have showed that disproportional reporting signals differ not only at the class level between anti-VEGF monoclonal antibodies and TKIs, but also among individual drugs (Ciccone et al., 2023). In FAERS, anti-VMAs exhibited significant disproportionate signals for all three endpoints (PAAD, PAA, and PAD), whereas SMV-TKIs were significantly associated with PAAD and PAD but not with PAA. In JADER, both subclasses displayed strong signals; however, anti-VMAs showed markedly elevated odds for PAA, while SMV-TKIs exhibited extraordinarily high odds for PAD. This subclass-specific divergence was further reinforced by our drug-specific analyses: ramucirumab yielded consistent PAA signals across both databases, whereas lenvatinib exhibited robust PAD associations. These observations suggested that although both drug subclasses contributed to the overall PAAD signal, they might have preferentially predisposed to distinct pathological phenotypes, namely aneurysmal dilation following exposure to anti-VMA agents and dissection following SMV-TKI treatment.

This subclass-specific pattern likely reflected differences in target selectivity and pathway inhibition depth. Anti-VMAs bound VEGF ligands and broadly suppressed VEGF-A-driven endothelial survival and nitric oxide production, promoting both medial degeneration and hypertension, which were pathological substrates that predisposed to both aneurysm formation and dissection (Lee et al., 2025; Gonzalez et al., 2025; Pérez-Gutiérrez and Ferrara, 2023). SMV-TKIs, by contrast, inhibited a wider panel of receptor tyrosine kinases, which might more profoundly compromise vascular smooth muscle cell viability and extracellular matrix homeostasis, thereby favoring dissection over aneurysmal dilation (Zhou et al., 2023; Ran et al., 2021). The phosphoinositide 3-kinase (PI3K)-protein kinase B (AKT) pathway inactivation and consequent upregulation of matrix metalloproteinase-9 (MMP-9) with concomitant downregulation of tissue inhibitor of metalloproteinase-1 (TIMP-1) represents a mechanism previously documented for VEGF inhibitors (Wang et al., 2022; Deng and Zhou, 2023). This regulatory pattern provided a biologically plausible explanation for the medial degradation that underlay both entities. Our data, therefore, added granularity to the emerging consensus that VEGF(R) inhibition produced arterial wall injury through alteration of the extracellular matrix, vasa vasorum rarefaction, and induction of medial degeneration, with the relative contribution of each process varying by drug class.

Time-to-onset analysis identified a distinct temporal pattern. In FAERS, AI-related PAAD was early-dominant, with 50% of events occurring within the first 29 days. PAD displayed a heterogeneous distribution with both early and late clusters. In JADER, the divergence was even more pronounced: PAA was early-dominant, whereas PAD was markedly right-skewed and late-dominant. Multivariable temporal analysis confirmed that the 0–29 days and ≥360 days windows carried the highest adjusted odds for PAAD and PAD in both cohorts, with the ≥360 days window yielding the most extreme estimates.

The early window likely captures AI-induced acute perturbations, most notably VEGF-inhibition-mediated hypertension, which could manifest within days of treatment initiation and, in susceptible arteries, precipitate dissection upon a substrate of pre-existing medical stress (le Noble et al., 2023; Palaskas et al., 2026). Indeed, poorly controlled blood pressure during AI therapy has been identified as a key precipitating factor for artery aneurysm or dissection (Sakalihasan et al., 2005; Clouse et al., 2004). The late window, conversely, might reflect the cumulative effect of chronic VEGF-pathway suppression on vascular wall remodeling (Zhao et al., 2004). Progressive medial degeneration, and matrix metalloproteinase-mediated extracellular matrix degradation progressively weaken the arterial wall, culminating in dissection or aneurysmal dilation months to years after exposure (Liu et al., 2023; Siafakas et al., 2007; Bararu-Bojan et al., 2024). This biphasic temporal signature carries direct clinical implications: arterial monitoring should not be confined to the treatment-initiation period but extended well beyond 1 year in patients receiving long-term AI therapy.

Notably, multivariable-adjusted analyses across both databases failed to identify hypertension as a predictor of PAAD. Accumulated case reports have documented artery aneurysm and dissection events following anti-VEGF therapy in normotensive patients without preexisting hypertension (Formiga and Fanelli, 2015). Similarly, a postmarketing pharmacovigilance study based on the FDA FAERS database analyzed 240 eligible cases and screened 15 events classified as “probable” according to the WHO-UMC causality assessment scale. These validated cases presented no major confounding factors for artery aneurysm or dissection, including uncontrolled hypertension (Cheng et al., 2021). These observations indicate that AI-associated arterial wall injury might occur independently of hypertensive vascular burden.

An unexpected observation was that age ≥70 years was associated with lower reporting odds of PAAD and PAD after adjustment. This counterintuitive finding was most plausibly explained by prescription bias and survivor effects rather than true biological protection. Elderly patients with advanced malignancy and pre-existing vascular comorbidity were less likely to be prescribed AIs, or were prescribed lower doses, generating a selection of healthier-than-average elderly individuals into the AI-exposed group. Non-healthcare-professional reporting was likewise associated with lower PAD odds, reflecting the well-described under-detection of asymptomatic or clinically subtle arterial events outside specialized oncological care.

Several key limitations should be acknowledged. First, all temporal analyses, including time-to-onset and Weibull modeling, were performed among a limited number of timestamp-complete case reports, especially in the JADER dataset and pharmacological subclass strata. Accordingly, the observed biphasic temporal patterns and subgroup-specific reporting trends are exploratory and hypothesis-generating rather than definitive conclusions, requiring further external validation. Second, although multivariable adjustment was applied, residual confounding remains inevitable in spontaneous pharmacovigilance data. Third, covariate information for hypertension, diabetes mellitus, quinolone use, and immunotherapy lacks explicit true-negative documentation in FAERS and JADER. We improved covariate capture by integrating both disease diagnosis and medication records; nevertheless, unreported entries were conservatively coded as absence in the primary analysis, which may misclassify underreported comorbidities or exposures as negative and introduce misclassification bias. The alternative assumption of treating all missing entries as positive cannot be statistically evaluated, as it produces zero-variance covariates that preclude regression modeling. Fourth, subclass-stratified analyses were restricted to SMV-TKIs and anti-VMAs, while individual drug-level analyses were underpowered due to limited event counts. Fifth, significant cross-database heterogeneity exists: FAERS and JADER represent distinct population sources with divergent reporting practices, limiting direct quantitative comparison and restricting generalizability. Sixth, anatomical stratification was limited by a high proportion of cases coded as “not otherwise specified” (NOS), which precluded precise localization of vascular events and might obscure territory-specific risk patterns. Finally, although the Benjamini-Hochberg FDR correction was applied to control type I error for primary analyses, exploratory temporal and stratified findings remain susceptible to multiple-testing bias and should be interpreted cautiously. Collectively, all outcomes in this study reflect disproportional reporting associations rather than established causal relationships.

## 5 Conclusion

In conclusion, this cross-database study characterized disproportionate reporting signals and temporal patterns of AI-related PAAD using FAERS and JADER datasets. Robust, cross-cohort signals for composite PAAD and PAD were identified, with amplified effect sizes in JADER, while PAA presented distinct cohort- and subclass-specific features. Notably, AI subclasses exhibited divergent vascular toxicity profiles: anti-VMAs increased reporting of PAAD, PAA, and PAD, whereas SMV-TKIs primarily enhanced PAAD and PAD signals, demonstrating subclass-specific adverse outcomes rather than uniform class-wide toxicity. Temporal analyses confirmed the onset pattern for AI-associated arterial events. PAAD and PAA predominantly emerged early after treatment initiation, while PAD showed marked delayed onset. Both the early (0–29 days) and long-term (≥360 days) exposure windows yielded elevated reporting odds, with substantially stronger late-phase signals suggestive of cumulative vascular injury from sustained VEGF pathway suppression. These findings indicated AI-related arterial adverse events could manifest throughout treatment, not merely in the initial therapeutic stage. In summary, this study provided refined real-world evidence for the association between AI exposure and PAAD, with clear subclass and temporal heterogeneity. Constrained by inherent biases of spontaneous reporting systems, these results represented statistical disproportionality signals rather than definitive causal relationships. Clinically, continuous long-term vascular surveillance was essential for AI-treated patients. Further prospective and mechanistic studies were required to validate these temporal toxicity patterns, clarify subclass-specific event burdens, and optimize individualized monitoring strategies.

## Data Availability

The raw data supporting the conclusions of this article will be made available by the authors, without undue reservation.
