# Supplementary material for: Angiogenesis inhibitors and the risk of peripheral artery aneurysm and dissection: a cross-database study
Source: Front Pharmacol. 2026 Sep 22;17:1932218. doi: 10.3389/fphar.2026.1932218 (PMC13638653; doi:10.3389/fphar.2026.1932218)
Supplement: Supplementary file 1 [file Supplementaryfile1.docx]

Supplementary Table 1. Baseline Characteristics in the JADER Cohort

| **Characteristics** | **Non**-**AI** | **AI** | ***P***-**value** | ***q***-**value** |
| --- | --- | --- | --- | --- |
| **JADER Cohort** | **N = 117,346** | **N = 19,414** |  |  |
| Age group (years) |  |  | <0.001 | <0.001 |
| < 70 | 56,361 (48.0%) | 10,297 (53.0%) |  |  |
| ≥ 70 | 60,985 (52.0%) | 9,117 (47.0%) |  |  |
| Gender |  |  | <0.001 | <0.001 |
| Male | 47,648 (40.6%) | 8,270 (42.6%) |  |  |
| Female | 69,698 (59.4%) | 11,144 (57.4%) |  |  |
| Weight group (kg) |  |  | <0.001 | <0.001 |
| < 70 | 61,811 (86.4%) | 7,734 (83.6%) |  |  |
| ≥ 70 | 9,755 (13.6%) | 1,520 (16.4%) |  |  |
| Missing | 45,780 (39.0%) | 10,160 (52.3%) |  |  |
| Hypertension | 26,757 (22.8%) | 5,403 (27.8%) | <0.001 | <0.001 |
| Diabetes | 14,933 (12.7%) | 2,098 (10.8%) | <0.001 | <0.001 |
| Quinolones | 3,638 (3.1%) | 182 (0.9%) | <0.001 | <0.001 |
| Immunotherapy | 35,116 (29.9%) | 7,574 (39.0%) | <0.001 | <0.001 |
| Reporters type |  |  | <0.001 | <0.001 |
| Health expert | 116,487 (99.3%) | 19,394 (99.9%) |  |  |
| Non-health expert | 205 (0.2%) | 9 (0.0%) |  |  |
| Missing | 654 (0.6%) | 11 (0.1%) |  |  |

JADER, Japanese Adverse Drug Event Report Database; AI, angiogenesis inhibitor; *q*-value, multiple-testing adjusted *P*-value.

Supplementary Table 2. Disproportionality Analysis of AI-Related PAAD in the JADER Cohort

| **Characteristic** | **Overall Events** | **PAAD Events** | **ROR (95% CI)** |
| --- | --- | --- | --- |
| **JADER Cohort** |  |  |  |
| PAAD |  |  | 8.465 (4.641, 15.441) |
| Non-AI | 216,080 | 18 |  |
| AI | 36,895 | 26 |  |
| PAA |  |  | 8.113 (3.975, 16.559) |
| Non-AI | 216,080 | 13 |  |
| AI | 36,895 | 18 |  |
| PAD |  |  | 9.372 (3.066, 28.651) |
| Non-AI | 216,080 | 5 |  |
| AI | 36,895 | 8 |  |
| **SMV-TKIs Subgroup** |  |  |  |
| PAAD |  |  | 6.533 (3.085, 13.834) |
| Non-AI | 216,080 | 18 |  |
| AI | 20,222 | 11 |  |
| PAA |  |  | 3.288 (1.072, 10.086) |
| Non-AI | 216,080 | 13 |  |
| AI | 20,222 | 4 |  |
| PAD |  |  | 14.964 (4.749, 47.154) |
| Non-AI | 216,080 | 5 |  |
| AI | 20,222 | 7 |  |
| **Anti-VMAs Subgroup** |  |  |  |
| PAAD |  |  | 10.809 (5.446, 21.451) |
| Non-AI | 216,080 | 18 |  |
| AI | 16,673 | 15 |  |
| PAA |  |  | 13.968 (6.564, 29.720) |
| Non-AI | 216,080 | 13 |  |
| AI | 16,673 | 14 |  |
| PAD |  |  | 2.592 (0.303, 22.189) |
| Non-AI | 216,080 | 5 |  |
| AI | 16,673 | 1 |  |

JADER, Japanese Adverse Drug Event Report Database; AI, angiogenesis inhibitor; SMV-TKIs, small-molecule VEGFR tyrosine kinase inhibitors; VEGFR, vascular endothelial growth factor receptor; Anti-VMAs, anti-VEGF monoclonal antibodies; PAAD, peripheral artery aneurysm and dissection; PAA, peripheral artery aneurysm; PAD, peripheral artery dissection; ROR, reporting odds ratio; CI, confidence interval.

Supplementary Table 3. Disproportionality Analysis of AI-Related PAAD for Individual Drugs

| **Drugs** | **Event Type** | **AI** | | **Non-AI** | | **ROR (95% CI)** |
| --- | --- | --- | --- | --- | --- | --- |
|  |  | **Overall Events** | **PAAD Events** | **Overall Events** | **PAAD Events** |  |
| **FAERS Cohort** |  |  |  |  |  |  |
| Aflibercept | PAD | 4,835 | 2 | 2,440,300 | 24 | 42.077 (9.941, 178.090) |
| Bevacizumab | PAA | 76,076 | 3 | 2,440,300 | 51 | 1.887 (0.411, 6.046) |
| Bevacizumab | PAD | 76,076 | 6 | 2,440,300 | 24 | 8.020 (1.010, 19.620) |
| Cabozantinib | PAA | 62,055 | 2 | 2,440,300 | 51 | 1.542 (0.294, 6.335) |
| Cabozantinib | PAD | 62,055 | 1 | 2,440,300 | 24 | 1.639 (0.168, 12.113) |
| Lenvatinib | PAA | 72,684 | 1 | 2,440,300 | 51 | 0.658 (0.115, 4.764) |
| Lenvatinib | PAD | 72,684 | 10 | 2,440,300 | 24 | 13.991 (1.504, 29.258) |
| Ramucirumab | PAA | 5,736 | 4 | 2,440,300 | 51 | 33.390 (1.658, 92.416) |
| Sunitinib | PAA | 53,947 | 2 | 2,440,300 | 51 | 1.774 (0.312, 7.287) |
| **JADER Cohort** |  |  |  |  |  |  |
| Bevacizumab | PAA | 12,910 | 7 | 216,080 | 13 | 9.017 (1.037, 22.604) |
| Bevacizumab | PAD | 12,910 | 1 | 216,080 | 5 | 3.348 (0.197, 28.657) |
| Fruquintinib | PAA | 510 | 1 | 216,080 | 13 | 32.653 (0.593, 250.083) |
| Lenvatinib | PAA | 11,848 | 3 | 216,080 | 13 | 4.210 (0.532, 14.774) |
| Lenvatinib | PAD | 11,848 | 7 | 216,080 | 5 | 25.547 (1.296, 80.506) |
| Ramucirumab | PAA | 3,662 | 7 | 216,080 | 13 | 31.831 (1.792, 79.830) |

FAERS, U.S. Food and Drug Administration Adverse Event Reporting System; JADER, Japanese Adverse Drug Event Report Database; AI, angiogenesis inhibitor; PAAD, peripheral artery aneurysm and dissection; PAA, peripheral artery aneurysm; PAD, peripheral artery dissection; ROR, reporting odds ratio; CI, confidence interval.

Supplementary Table 4. Disproportionality Analysis of AI-Related PAAD in Anatomical Distribution

| **Anatomical Distribution** | **AI** | | **Non-AI** | | **ROR (95% CI)** |
| --- | --- | --- | --- | --- | --- |
|  | **Overall Events** | **PAAD Events** | **Overall Events** | **PAAD Events** |  |
| **FAERS Cohort** |  |  |  |  |  |
| Peripheral Artery Dissection, NOS | 307,853 | 13 | 2,440,300 | 8 | 12.882 (5.339, 31.080) |
| Peripheral Artery Aneurysm, NOS | 307,853 | 8 | 2,440,300 | 33 | 1.922 (0.613, 4.160) |
| Femoral Artery Dissection | 307,853 | 1 | 2,440,300 | 0 |  |
| Hepatic Artery Aneurysm | 307,853 | 3 | 2,440,300 | 2 | 11.890 (0.490, 71.162) |
| Intratumoral Artery Aneurysm | 307,853 | 1 | 2,440,300 | 0 |  |
| Renal Artery Dissection | 307,853 | 2 | 2,440,300 | 1 | 15.854 (0.301, 174.847) |
| Subclavian Artery Dissection | 307,853 | 1 | 2,440,300 | 0 |  |
| Superior Mesenteric Artery Dissection | 307,853 | 2 | 2,440,300 | 1 | 15.854 (0.301, 174.847) |
| **JADER Cohort** |  |  |  |  |  |
| Peripheral Artery Dissection, NOS | 36,895 | 6 | 216,080 | 3 | 11.715 (0.728, 46.845) |
| Peripheral Artery Aneurysm, NOS | 36,895 | 13 | 216,080 | 13 | 5.858 (0.999, 12.638) |
| Superior Mesenteric Artery Dissection | 36,895 | 2 | 216,080 | 1 | 11.714 (0.264, 129.191) |
| Hepatic Artery Aneurysm | 36,895 | 4 | 216,080 | 0 |  |
| Intratumoral Artery Aneurysm | 36,895 | 1 | 216,080 | 0 |  |

FAERS, U.S. Food and Drug Administration Adverse Event Reporting System; JADER, Japanese Adverse Drug Event Report Database; AI, angiogenesis inhibitor; PAAD, peripheral artery aneurysm and dissection; NOS, not otherwise specified; ROR, reporting odds ratio; CI, confidence interval.

Supplementary Table 5. Time-to-Onset Analysis of AI-Related PAAD by Weibull Distribution Analysis in the JADER Cohort

| **Characteristics** | **TTO (days)** | | **Weibull distribution** | | |
| --- | --- | --- | --- | --- | --- |
|  | **Case reports** | **Median (d) (IQR)** | **Scale parameter: log(α) (95% CI)** | **Shape parameter: β (95% CI)** | **Pattern** |
| **JADER Cohort** |  |  |  |  |  |
| PAAD | 22 | 50.0 (27.0, 151.0) | 13.026 (10.758 - 15.294) | 0.818 (0.611 - 1.094) | Early trend |
| PAA | 15 | 43.0 (23.0, 84.0) | 16.082 (11.674 - 20.49) | 0.622 (0.419 - 0.923) | Early dominant |
| PAD | 8 | 853.0 (41.5, 943.0) | 11.227 (9.432 - 13.022) | 1.276 (0.907 - 1.794) | Late trend |
| **SMV-TKIs Subgroup** |  |  |  |  |  |
| PAAD | 10 | 63.0 (23.0, 859.0) | 11.781 (9.226 - 14.335) | 0.992 (0.664 - 1.482) | Near constant |
| PAA | 4 | 25.5 (16.5, 49.5) | 17.963 (7.603 - 28.324) | 0.580 (0.264 - 1.274) | Early trend |
| PAD | 7 | 847.0 (28.0, 859.0) | 10.758 (8.634 - 12.882) | 1.268 (0.827 - 1.944) | Late trend |
| **Anti-VMAs Subgroup** |  |  |  |  |  |
| PAAD | 12 | 44.0 (34.0, 103.0) | 14.216 (10.386 - 18.046) | 0.699 (0.458 - 1.065) | Early trend |
| PAA | 11 | 43.0 (27.0, 94.0) | 15.398 (10.625 - 20.172) | 0.626 (0.395 - 0.990) | Early dominant |
| PAD | 1 | 1,174.0 (1,174.0, 1,174.0) | 11.109 (8.850 - 13.369) | 1.665 (0.950 - 2.917) | Late trend |

JADER, Japanese Adverse Drug Event Report Database; AI, angiogenesis inhibitor; SMV-TKIs, small-molecule VEGFR tyrosine kinase inhibitors; VEGFR, vascular endothelial growth factor receptor; Anti-VMAs, anti-VEGF monoclonal antibodies; PAAD, peripheral artery aneurysm and dissection; PAA, peripheral artery aneurysm; PAD, peripheral artery dissection; TTO, time-to-onset; IQR, interquartile range; CI, confidence interval.

Supplementary Table 6. Univariate Logistic Regression Analysis of Factors Associated with AI-Related PAAD in the FAERS Cohort

| **Characteristics** | **PAAD** | | | **PAA** | | | **PAD** | | |
| --- | --- | --- | --- | --- | --- | --- | --- | --- | --- |
| **FAERS Cohort** | **cOR (95% CI)** | ***P***-**value** | ***q***-**value** | **cOR (95% CI)** | ***P***-**value** | ***q***-**value** | **cOR (95% CI)** | ***P***-**value** | ***q***-**value** |
| Age group (years) |  |  |  |  |  |  |  |  |  |
| < 70 years | Reference |  |  | Reference |  |  | Reference |  |  |
| ≥ 70 years | 0.734 (0.484, 1.095) | 0.131 | 0.262 | 1.113 (0.670, 1.833) | 0.675 | 0.942 | 0.354 (0.156, 0.718) | 0.003 | 0.008 |
| Gender |  |  |  |  |  |  |  |  |  |
| Male | Reference |  |  | Reference |  |  | Reference |  |  |
| Female | 1.085 (0.737, 1.601) | 0.679 | 0.754 | 0.854 (0.515, 1.406) | 0.535 | 0.892 | 1.552 (0.843, 2.937) | 0.159 | 0.199 |
| Diabetes |  |  |  |  |  |  |  |  |  |
| No | Reference |  |  | Reference |  |  | Reference |  |  |
| Yes | 0.624 (0.254, 1.274) | 0.212 | 0.353 | 0.902 (0.332, 1.979) | 0.814 | 0.942 | 0.343 (0.039, 1.275) | 0.126 | 0.179 |
| Hypertension |  |  |  |  |  |  |  |  |  |
| No | Reference |  |  | Reference |  |  | Reference |  |  |
| Yes | 1.224 (0.727, 1.959) | 0.432 | 0.549 | 1.599 (0.857, 2.805) | 0.134 | 0.447 | 0.789 (0.286, 1.781) | 0.595 | 0.595 |
| AI |  |  |  |  |  |  |  |  |  |
| No | Reference |  |  | Reference |  |  | Reference |  |  |
| Yes | 3.925 (2.535, 5.929) | <0.001 | <0.001 | 2.355 (1.212, 4.238) | 0.013 | 0.067 | 7.343 (3.945, 13.468) | <0.001 | <0.001 |
| AI type |  |  |  |  |  |  |  |  |  |
| None | Reference |  |  | Reference |  |  | Reference |  |  |
| SMV-TKIs | 3.314 (1.905, 5.450) | <0.001 | <0.001 | 1.525 (0.559, 3.371) | 0.375 | 0.749 | 7.302 (3.564, 14.254) | <0.001 | <0.001 |
| Anti-VMAs | 5.421 (2.902, 9.367) | <0.001 | <0.001 | 4.410 (1.880, 8.932) | 0.002 | 0.015 | 8.050 (3.093, 18.120) | <0.001 | <0.001 |
| Immunotherapy |  |  |  |  |  |  |  |  |  |
| No | Reference |  |  | Reference |  |  | Reference |  |  |
| Yes | 1.052 (0.590, 1.751) | 0.855 | 0.855 | 0.578 (0.213, 1.268) | 0.186 | 0.464 | 1.979 (0.938, 3.849) | 0.072 | 0.119 |
| Quinolones |  |  |  |  |  |  |  |  |  |
| No | Reference |  |  | Reference |  |  | Reference |  |  |
| Yes | 0.561 (0.064, 2.029) | 0.439 | 0.549 | 0.942 (0.107, 3.448) | 0.942 | 0.942 | 0.458 (0.004, 3.200) | 0.53 | 0.589 |
| Reporter type |  |  |  |  |  |  |  |  |  |
| Health expert | Reference |  |  | Reference |  |  | Reference |  |  |
| Non-health expert | 0.631 (0.365, 1.028) | 0.065 | 0.163 | 0.950 (0.508, 1.669) | 0.863 | 0.942 | 0.277 (0.076, 0.721) | 0.006 | 0.012 |

cOR, crude odds ratio; CI, confidence interval; *q*-value, multiple-testing adjusted *P*-value; FAERS, U.S. Food and Drug Administration Adverse Event Reporting System; AI, angiogenesis inhibitor; SMV-TKIs, small-molecule VEGFR tyrosine kinase inhibitors; VEGFR, vascular endothelial growth factor receptor; Anti-VMAs, anti-VEGF monoclonal antibodies; PAAD, peripheral artery aneurysm and dissection; PAA, peripheral artery aneurysm; PAD, peripheral artery dissection.

Supplementary Table 7. Multivariate Logistic Regression Analysis of Factors Associated with AI-Related PAAD in the FAERS Cohort: Subgroup Analysis

| **Characteristics** | **PAAD** | | | **PAA** | | | **PAD** | | |
| --- | --- | --- | --- | --- | --- | --- | --- | --- | --- |
| **FAERS Cohort** | **aOR (95% CI)** | ***P***-**value** | ***q***-**value** | **aOR (95% CI)** | ***P***-**value** | ***q***-**value** | **aOR (95% CI)** | ***P***-**value** | ***q***-**value** |
| Age group (years) |  |  |  |  |  |  |  |  |  |
| < 70 years | Reference |  |  | Reference |  |  | Reference |  |  |
| ≥ 70 years | 0.773 (0.507, 1.160) | 0.217 | 0.325 | 1.108 (0.659, 1.847) | 0.695 | 0.893 | 0.400 (0.175, 0.813) | 0.010 | 0.023 |
| Gender |  |  |  |  |  |  |  |  |  |
| Male | Reference |  |  | Reference |  |  | Reference |  |  |
| Female | 1.064 (0.718, 1.577) | 0.757 | 0.757 | 0.814 (0.486, 1.352) | 0.426 | 0.639 | 1.551 (0.838, 2.949) | 0.163 | 0.294 |
| Diabetes |  |  |  |  |  |  |  |  |  |
| No | Reference |  |  | Reference |  |  | Reference |  |  |
| Yes | 0.488 (0.177, 1.092) | 0.084 | 0.190 | 0.582 (0.184, 1.441) | 0.261 | 0.558 | 0.417 (0.046, 1.651) | 0.246 | 0.369 |
| Hypertension |  |  |  |  |  |  |  |  |  |
| No | Reference |  |  | Reference |  |  | Reference |  |  |
| Yes | 1.411 (0.819, 2.313) | 0.206 | 0.325 | 1.843 (0.954, 3.342) | 0.068 | 0.261 | 0.905 (0.319, 2.105) | 0.830 | 0.830 |
| AI type |  |  |  |  |  |  |  |  |  |
| None | Reference |  |  | Reference |  |  | Reference |  |  |
| SMV-TKIs | 3.593 (2.032, 6.026) | <0.001 | <0.001 | 1.639 (0.593, 3.694) | 0.310 | 0.558 | 8.180 (3.854, 16.573) | <0.001 | <0.001 |
| Anti-VMAs | 5.090 (2.712, 8.853) | <0.001 | <0.001 | 4.709 (1.993, 9.644) | 0.001 | 0.010 | 6.504 (2.489, 14.722) | <0.001 | 0.002 |
| Immunotherapy |  |  |  |  |  |  |  |  |  |
| No | Reference |  |  | Reference |  |  | Reference |  |  |
| Yes | 0.773 (0.428, 1.309) | 0.351 | 0.452 | 0.492 (0.179, 1.098) | 0.087 | 0.261 | 1.165 (0.536, 2.346) | 0.685 | 0.771 |
| Quinolones |  |  |  |  |  |  |  |  |  |
| No | Reference |  |  | Reference |  |  | Reference |  |  |
| Yes | 0.605 (0.069, 2.206) | 0.508 | 0.571 | 0.914 (0.103, 3.400) | 0.912 | 0.912 | 0.576 (0.005, 4.086) | 0.672 | 0.771 |
| Reporter type |  |  |  |  |  |  |  |  |  |
| Health expert | Reference |  |  | Reference |  |  | Reference |  |  |
| Non-health expert | 0.606 (0.348, 0.996) | 0.048 | 0.144 | 0.950 (0.505, 1.682) | 0.865 | 0.912 | 0.250 (0.068, 0.661) | 0.003 | 0.010 |

aOR, adjusted odds ratio; CI, confidence interval; *q*-value, multiple-testing adjusted *P*-value; FAERS, U.S. Food and Drug Administration Adverse Event Reporting System; AI, angiogenesis inhibitor; SMV-TKIs, small-molecule VEGFR tyrosine kinase inhibitors; VEGFR, vascular endothelial growth factor receptor; Anti-VMAs, anti-VEGF monoclonal antibodies; PAAD, peripheral artery aneurysm and dissection; PAA, peripheral artery aneurysm; PAD, peripheral artery dissection.

Supplementary Table 8. Univariate Logistic Regression Analysis of Factors Associated with AI-Related PAAD in the JADER Cohort

| **Characteristics** | **PAAD** | | | **PAA** | | | **PAD** | | |
| --- | --- | --- | --- | --- | --- | --- | --- | --- | --- |
| **JADER Cohort** | **cOR (95% CI)** | ***P***-**value** | ***q***-**value** | **cOR (95% CI)** | ***P***-**value** | ***q***-**value** | **cOR (95% CI)** | ***P***-**value** | ***q***-**value** |
| Age group (years) |  |  |  |  |  |  |  |  |  |
| < 70 years | Reference |  |  | Reference |  |  | Reference |  |  |
| ≥ 70 years | 0.306 (0.139, 0.613) | <0.001 | 0.002 | 0.414 (0.175, 0.899) | 0.025 | 0.069 | 0.124 (0.013, 0.519) | 0.002 | 0.009 |
| Gender |  |  |  |  |  |  |  |  |  |
| Male | Reference |  |  | Reference |  |  | Reference |  |  |
| Female | 0.766 (0.408, 1.449) | 0.409 | 0.499 | 1.153 (0.545, 2.564) | 0.714 | 0.729 | 0.366 (0.106, 1.107) | 0.075 | 0.207 |
| Diabetes |  |  |  |  |  |  |  |  |  |
| No | Reference |  |  | Reference |  |  | Reference |  |  |
| Yes | 0.481 (0.099, 1.431) | 0.213 | 0.390 | 0.689 (0.140, 2.103) | 0.554 | 0.678 | 0.281 (0.002, 2.132) | 0.279 | 0.384 |
| Hypertension |  |  |  |  |  |  |  |  |  |
| No | Reference |  |  | Reference |  |  | Reference |  |  |
| Yes | 1.534 (0.757, 2.945) | 0.225 | 0.354 | 1.670 (0.731, 3.570) | 0.213 | 0.293 | 1.198 (0.299, 3.774) | 0.776 | 0.827 |
| AI |  |  |  |  |  |  |  |  |  |
| No | Reference |  |  | Reference |  |  | Reference |  |  |
| Yes | 8.250 (4.392, 15.824) | <0.001 | <0.001 | 7.500 (3.564, 16.101) | <0.001 | <0.001 | 11.421 (3.779, 39.630) | <0.001 | <0.001 |
| AI type |  |  |  |  |  |  |  |  |  |
| None | Reference |  |  | Reference |  |  | Reference |  |  |
| SMV-TKIs | 7.497 (3.347, 16.052) | <0.001 | <0.001 | 4.239 (1.277, 11.664) | 0.021 | 0.069 | 19.631 (6.251, 69.336) | <0.001 | <0.001 |
| Anti-VMAs | 9.422 (4.424, 19.572) | <0.001 | <0.001 | 11.442 (5.058, 25.652) | <0.001 | <0.001 | 4.141 (0.415, 22.355) | 0.187 | 0.343 |
| Immunotherapy |  |  |  |  |  |  |  |  |  |
| No | Reference |  |  | Reference |  |  | Reference |  |  |
| Yes | 0.710 (0.323, 1.420) | 0.344 | 0.473 | 0.422 (0.133, 1.054) | 0.066 | 0.121 | 1.616 (0.506, 4.838) | 0.400 | 0.489 |
| Quinolones |  |  |  |  |  |  |  |  |  |
| No | Reference |  |  | Reference |  |  | Reference |  |  |
| Yes | 0.452 (0.004, 3.168) | 0.522 | 0.522 | 0.633 (0.005, 4.502) | 0.729 | 0.729 | 1.392 (0.011, 10.556) | 0.827 | 0.827 |
| Reporter type |  |  |  |  |  |  |  |  |  |
| Health expert | Reference |  |  | Reference |  |  | Reference |  |  |
| Non-health expert | 8.444 (0.067, 59.625) | 0.264 | 0.415 | 11.950 (0.094, 85.735) | 0.212 | 0.334 | 25.337 (0.198, 193.242) | 0.133 | 0.293 |
| Missing | 8.177 (0.920, 30.595) | 0.057 | 0.125 | 11.573 (1.293, 44.217) | 0.033 | 0.073 | 8.166 (0.064, 62.036) | 0.272 | 0.384 |

cOR, crude odds ratio; CI, confidence interval; *q*-value, multiple-testing adjusted *P*-value; JADER, Japanese Adverse Drug Event Report Database; AI, angiogenesis inhibitor; SMV-TKIs, small-molecule VEGFR tyrosine kinase inhibitors; VEGFR, vascular endothelial growth factor receptor; Anti-VMAs, anti-VEGF monoclonal antibodies; PAAD, peripheral artery aneurysm and dissection; PAA, peripheral artery aneurysm; PAD, peripheral artery dissection.

Supplementary Table 9. Multivariate Logistic Regression Analysis of Factors Associated with AI-Related PAAD in the JADER Cohort: Subgroup Analysis

| **Characteristics** | **PAAD** | | | **PAA** | | | **PAD** | | |
| --- | --- | --- | --- | --- | --- | --- | --- | --- | --- |
| **JADER Cohort** | **aOR (95% CI)** | ***P***-**value** | ***q***-**value** | **aOR (95% CI)** | ***P***-**value** | ***q***-**value** | **aOR (95% CI)** | ***P***-**value** | ***q***-**value** |
| Age group (years) |  |  |  |  |  |  |  |  |  |
| < 70 years | Reference |  |  | Reference |  |  | Reference |  |  |
| ≥ 70 years | 0.327 (0.148, 0.662) | 0.001 | 0.005 | 0.434 (0.181, 0.955) | 0.038 | 0.094 | 0.142 (0.015, 0.602) | 0.006 | 0.028 |
| Gender |  |  |  |  |  |  |  |  |  |
| Male | Reference |  |  | Reference |  |  | Reference |  |  |
| Female | 0.916 (0.485, 1.740) | 0.786 | 0.786 | 1.384 (0.648, 3.101) | 0.405 | 0.506 | 0.483 (0.138, 1.477) | 0.205 | 0.409 |
| Diabetes |  |  |  |  |  |  |  |  |  |
| No | Reference |  |  | Reference |  |  | Reference |  |  |
| Yes | 0.486 (0.097, 1.514) | 0.238 | 0.297 | 0.643 (0.127, 2.087) | 0.498 | 0.554 | 0.351 (0.003, 2.944) | 0.406 | 0.581 |
| Hypertension |  |  |  |  |  |  |  |  |  |
| No | Reference |  |  | Reference |  |  | Reference |  |  |
| Yes | 1.837 (0.887, 3.599) | 0.099 | 0.198 | 1.916 (0.815, 4.207) | 0.131 | 0.218 | 1.562 (0.383, 5.034) | 0.499 | 0.623 |
| AI type |  |  |  |  |  |  |  |  |  |
| None | Reference |  |  | Reference |  |  | Reference |  |  |
| SMV-TKIs | 7.641 (3.346, 16.734) | <0.001 | <0.001 | 4.612 (1.373, 12.928) | 0.016 | 0.059 | 18.075 (5.298, 68.336) | <0.001 | <0.001 |
| Anti-VMAs | 8.352 (3.887, 17.537) | <0.001 | <0.001 | 10.562 (4.606, 24.094) | <0.001 | <0.001 | 3.396 (0.339, 18.530) | 0.249 | 0.414 |
| Immunotherapy |  |  |  |  |  |  |  |  |  |
| No | Reference |  |  | Reference |  |  | Reference |  |  |
| Yes | 0.613 (0.275, 1.253) | 0.186 | 0.279 | 0.401 (0.126, 1.014) | 0.054 | 0.107 | 0.864 (0.248, 2.828) | 0.810 | 0.810 |
| Quinolones |  |  |  |  |  |  |  |  |  |
| No | Reference |  |  | Reference |  |  | Reference |  |  |
| Yes | 0.567 (0.004, 4.051) | 0.663 | 0.736 | 0.711 (0.006, 5.154) | 0.801 | 0.801 | 2.299 (0.018, 19.062) | 0.613 | 0.681 |
| Reporter type |  |  |  |  |  |  |  |  |  |
| Health expert | Reference |  |  | Reference |  |  | Reference |  |  |
| Non-health expert | 13.648 (0.107, 99.576) | 0.196 | 0.279 | 19.221 (0.150, 144.241) | 0.158 | 0.226 | 38.351 (0.295, 318.373) | 0.104 | 0.347 |
| Missing | 13.556 (1.502, 53.050) | 0.026 | 0.065 | 17.452 (1.912, 70.576) | 0.018 | 0.059 | 16.653 (0.127, 143.918) | 0.176 | 0.409 |

aOR, adjusted odds ratio; CI, confidence interval; *q*-value, multiple-testing adjusted *P*-value; JADER, Japanese Adverse Drug Event Report Database; AI, Angiogenesis Inhibitor; SMV-TKIs, small-molecule VEGFR tyrosine kinase inhibitors; VEGFR, vascular endothelial growth factor receptor; Anti-VMAs, anti-VEGF monoclonal antibodies; PAAD, peripheral artery aneurysm and dissection; PAA, peripheral artery aneurysm; PAD, peripheral artery dissection.

Supplementary Table 10. Multivariate Temporal Logistic Regression Analysis of AI-Related PAAD in the FAERS Cohort

| **Characteristics** | **PAAD** | | | **PAA** | | | **PAD** | | |
| --- | --- | --- | --- | --- | --- | --- | --- | --- | --- |
| **FAERS Cohort** | **aOR (95% CI)** | ***P***-**value** | ***q***-**value1** | **aOR (95% CI)** | ***P***-**value** | ***q***-**value1** | **aOR (95% CI)** | ***P***-**value** | ***q***-**value1** |
| Age group (years) |  |  |  |  |  |  |  |  |  |
| < 70 years | Reference |  |  | Reference |  |  | Reference |  |  |
| ≥ 70 years | 0.774 (0.508, 1.161) | 0.219 | 0.348 | 1.104 (0.657, 1.839) | 0.705 | 0.769 | 0.406 (0.178, 0.826) | 0.012 | 0.028 |
| Gender |  |  |  |  |  |  |  |  |  |
| Male | Reference |  |  | Reference |  |  | Reference |  |  |
| Female | 1.076 (0.728, 1.595) | 0.712 | 0.712 | 0.836 (0.499, 1.388) | 0.489 | 0.754 | 1.539 (0.833, 2.923) | 0.170 | 0.292 |
| Diabetes |  |  |  |  |  |  |  |  |  |
| No | Reference |  |  | Reference |  |  | Reference |  |  |
| Yes | 0.489 (0.178, 1.093) | 0.085 | 0.169 | 0.573 (0.181, 1.419) | 0.247 | 0.493 | 0.422 (0.046, 1.666) | 0.253 | 0.337 |
| Hypertension |  |  |  |  |  |  |  |  |  |
| No | Reference |  |  | Reference |  |  | Reference |  |  |
| Yes | 1.385 (0.803, 2.272) | 0.232 | 0.348 | 1.808 (0.936, 3.280) | 0.076 | 0.230 | 0.892 (0.314, 2.078) | 0.806 | 0.806 |
| Immunotherapy |  |  |  |  |  |  |  |  |  |
| No | Reference |  |  | Reference |  |  | Reference |  |  |
| Yes | 0.775 (0.429, 1.311) | 0.355 | 0.426 | 0.481 (0.175, 1.074) | 0.077 | 0.230 | 1.240 (0.575, 2.477) | 0.565 | 0.678 |
| Quinolones |  |  |  |  |  |  |  |  |  |
| No | Reference |  |  | Reference |  |  | Reference |  |  |
| Yes | 0.617 (0.070, 2.252) | 0.527 | 0.575 | 0.938 (0.106, 3.491) | 0.938 | 0.938 | 0.576 (0.005, 4.086) | 0.672 | 0.733 |
| Reporter type |  |  |  |  |  |  |  |  |  |
| Health expert | Reference |  |  | Reference |  |  | Reference |  |  |
| Non-health expert | 0.582 (0.336, 0.953) | 0.031 | 0.123 | 0.881 (0.470, 1.553) | 0.672 | 0.769 | 0.255 (0.069, 0.670) | 0.003 | 0.014 |
| Exposure windows (days) |  |  |  |  |  |  |  |  |  |
| Nonexposure | Reference |  |  | Reference |  |  | Reference |  |  |
| 0-29 | 4.372 (2.415, 7.425) | <0.001 | <0.001 | 3.248 (1.373, 6.654) | 0.010 | 0.117 | 6.940 (2.942, 14.890) | <0.001 | <0.001 |
| 30-89 | 2.967 (0.972, 6.923) | 0.055 | 0.133 | 1.498 (0.169, 5.572) | 0.647 | 0.769 | 6.901 (1.827, 19.038) | 0.008 | 0.023 |
| 90-239 | 1.967 (0.407, 5.717) | 0.343 | 0.426 | 1.842 (0.208, 6.857) | 0.503 | 0.754 | 3.408 (0.378, 13.294) | 0.220 | 0.330 |
| 240-359 | 4.798 (0.993, 13.965) | 0.051 | 0.133 | 4.398 (0.495, 16.401) | 0.148 | 0.356 | 8.506 (0.942, 33.230) | 0.055 | 0.110 |
| ≥360 | 11.654 (4.998, 23.341) | <0.001 | <0.001 | 5.751 (1.182, 17.009) | 0.034 | 0.202 | 26.271 (9.199, 62.769) | <0.001 | <0.001 |

FAERS, U.S. Food and Drug Administration Adverse Event Reporting System; AI, angiogenesis inhibitor; aOR, adjusted odds ratio; CI, confidence interval; *q*-value, multiple-testing adjusted *P*-value; PAAD, peripheral artery aneurysm and dissection; PAA, peripheral artery aneurysm; PAD, peripheral artery dissection.

Supplementary Table 11. Multivariate Temporal Logistic Regression Analysis of AI-Associated PAAD in FAERS Cohort: Subgroup Analysis

| **Characteristics** | **PAAD** | | | **PAA** | | | **PAD** | | |
| --- | --- | --- | --- | --- | --- | --- | --- | --- | --- |
| **FAERS Cohort** | **aOR (95% CI)** | ***P***-**value** | ***q***-**value1** | **aOR (95% CI)** | ***P***-**value** | ***q***-**value1** | **aOR (95% CI)** | ***P***-**value** | ***q***-**value1** |
| Age group (years) |  |  |  |  |  |  |  |  |  |
| < 70 years | Reference |  |  | Reference |  |  | Reference |  |  |
| ≥ 70 years | 0.776 (0.509, 1.165) | 0.224 | 0.339 | 1.110 (0.660, 1.850) | 0.691 | 0.904 | 0.403 (0.177, 0.821) | 0.011 | 0.038 |
| Gender |  |  |  |  |  |  |  |  |  |
| Male | Reference |  |  | Reference |  |  | Reference |  |  |
| Female | 1.065 (0.719, 1.580) | 0.752 | 0.752 | 0.815 (0.486, 1.355) | 0.430 | 0.610 | 1.553 (0.838, 2.956) | 0.163 | 0.252 |
| Diabetes |  |  |  |  |  |  |  |  |  |
| No | Reference |  |  | Reference |  |  | Reference |  |  |
| Yes | 0.491 (0.178, 1.097) | 0.087 | 0.164 | 0.582 (0.184, 1.444) | 0.263 | 0.446 | 0.419 (0.046, 1.654) | 0.248 | 0.352 |
| Hypertension |  |  |  |  |  |  |  |  |  |
| No | Reference |  |  | Reference |  |  | Reference |  |  |
| Yes | 1.398 (0.810, 2.295) | 0.219 | 0.339 | 1.842 (0.953, 3.342) | 0.068 | 0.241 | 0.880 (0.310, 2.054) | 0.784 | 0.784 |
| Immunotherapy |  |  |  |  |  |  |  |  |  |
| No | Reference |  |  | Reference |  |  | Reference |  |  |
| Yes | 0.791 (0.438, 1.339) | 0.396 | 0.518 | 0.497 (0.181, 1.109) | 0.092 | 0.241 | 1.208 (0.556, 2.432) | 0.617 | 0.723 |
| Quinolones |  |  |  |  |  |  |  |  |  |
| No | Reference |  |  | Reference |  |  | Reference |  |  |
| Yes | 0.609 (0.069, 2.220) | 0.514 | 0.624 | 0.919 (0.104, 3.418) | 0.918 | 0.955 | 0.580 (0.005, 4.113) | 0.676 | 0.723 |
| Reporter type |  |  |  |  |  |  |  |  |  |
| Health expert | Reference |  |  | Reference |  |  | Reference |  |  |
| Non-health expert | 0.597 (0.343, 0.982) | 0.042 | 0.089 | 0.943 (0.501, 1.670) | 0.846 | 0.955 | 0.245 (0.066, 0.647) | 0.003 | 0.012 |
| Exposure windows (days) | |  |  |  |  |  |  |  |  |
| Nonexposure | Reference |  |  | Reference |  |  | Reference |  |  |
| SMV-TKIs |  |  |  |  |  |  |  |  |  |
| 0-29 | 4.158 (1.956, 7.888) | <0.001 | 0.003 | 2.225 (0.606, 5.807) | 0.199 | 0.376 | 8.933 (3.351, 20.745) | <0.001 | <0.001 |
| 30-89 | 2.368 (0.490, 6.907) | 0.239 | 0.339 | 0.687 (0.005, 4.825) | 0.779 | 0.946 | 7.588 (1.513, 23.954) | 0.019 | 0.053 |
| 90-239 | 0.619 (0.005, 4.301) | 0.714 | 0.752 | 0.924 (0.007, 6.505) | 0.955 | 0.955 | 1.918 (0.015, 14.080) | 0.681 | 0.723 |
| 240-359 | 7.461 (1.539, 21.847) | 0.018 | 0.060 | 6.444 (0.724, 24.207) | 0.083 | 0.241 | 14.512 (1.599, 57.500) | 0.024 | 0.058 |
| ≥360 | 11.275 (3.676, 26.508) | <0.001 | 0.003 | 5.260 (0.591, 19.724) | 0.113 | 0.241 | 29.289 (7.680, 82.066) | <0.001 | <0.001 |
| Anti-VMAs |  |  |  |  |  |  |  |  |  |
| 0-29 | 5.074 (2.042, 10.532) | 0.001 | 0.006 | 5.908 (1.916, 14.044) | 0.004 | 0.072 | 5.101 (1.029, 15.755) | 0.047 | 0.093 |
| 30-89 | 5.309 (1.101, 15.384) | 0.040 | 0.089 | 5.251 (0.593, 19.492) | 0.113 | 0.241 | 8.600 (0.957, 33.245) | 0.054 | 0.093 |
| 90-239 | 5.311 (1.101, 15.394) | 0.040 | 0.089 | 5.336 (0.602, 19.832) | 0.110 | 0.241 | 8.469 (0.943, 32.739) | 0.055 | 0.093 |
| 240-359 | 2.640 (0.021, 18.269) | 0.555 | 0.629 | 4.444 (0.035, 31.166) | 0.399 | 0.610 | 6.940 (0.055, 50.150) | 0.300 | 0.393 |
| ≥360 | 14.101 (3.895, 35.791) | <0.001 | 0.003 | 9.688 (1.094, 35.938) | 0.043 | 0.241 | 28.098 (5.670, 86.759) | <0.001 | 0.004 |

FAERS, U.S. Food and Drug Administration Adverse Event Reporting System; AI, angiogenesis inhibitor; aOR, adjusted odds ratio; CI, confidence interval; *q*-value, multiple-testing adjusted *P*-value; SMV-TKIs, small-molecule VEGFR tyrosine kinase inhibitors; VEGFR, vascular endothelial growth factor receptor; Anti-VMAs, anti-VEGF monoclonal antibodies; PAAD, peripheral artery aneurysm and dissection; PAA, peripheral artery aneurysm; PAD, peripheral artery dissection.

Supplementary Table 12. Multivariate Temporal Logistic Regression Analysis of AI-Related PAAD in the JADER Cohort

| **Characteristics** | **PAAD** | **PAAD** | **PAAD** | **PAA** | **PAA** | **PAA** | **PAD** | **PAD** | **PAD** |
| --- | --- | --- | --- | --- | --- | --- | --- | --- | --- |
| **JADER Cohort** | **aOR (95% CI)** | ***P***-**value** | ***q***-**value1** | **aOR (95% CI)** | ***P***-**value** | ***q***-**value1** | **aOR (95% CI)** | ***P***-**value** | ***q***-**value1** |
| Age group (years) |  |  |  |  |  |  |  |  |  |
| < 70 years | Reference |  |  | Reference |  |  | Reference |  |  |
| ≥ 70 years | 0.323 (0.146, 0.654) | 0.001 | 0.004 | 0.405 (0.170, 0.889) | 0.024 | 0.066 | 0.153 (0.016, 0.654) | 0.008 | 0.055 |
| Gender |  |  |  |  |  |  |  |  |  |
| Male | Reference |  |  | Reference |  |  | Reference |  |  |
| Female | 0.902 (0.478, 1.713) | 0.75 | 0.75 | 1.303 (0.612, 2.913) | 0.497 | 0.585 | 0.477 (0.137, 1.448) | 0.194 | 0.36 |
| Diabetes |  |  |  |  |  |  |  |  |  |
| No | Reference |  |  | Reference |  |  | Reference |  |  |
| Yes | 0.493 (0.099, 1.538) | 0.249 | 0.359 | 0.647 (0.127, 2.099) | 0.504 | 0.585 | 0.344 (0.003, 2.911) | 0.398 | 0.556 |
| Hypertension |  |  |  |  |  |  |  |  |  |
| No | Reference |  |  | Reference |  |  | Reference |  |  |
| Yes | 1.810 (0.873, 3.549) | 0.107 | 0.232 | 1.922 (0.817, 4.220) | 0.129 | 0.28 | 1.552 (0.380, 5.010) | 0.505 | 0.596 |
| Immunotherapy |  |  |  |  |  |  |  |  |  |
| No | Reference |  |  | Reference |  |  | Reference |  |  |
| Yes | 0.617 (0.280, 1.242) | 0.183 | 0.307 | 0.354 (0.112, 0.890) | 0.026 | 0.066 | 1.577 (0.490, 4.766) | 0.428 | 0.556 |
| Quinolones |  |  |  |  |  |  |  |  |  |
| No | Reference |  |  | Reference |  |  | Reference |  |  |
| Yes | 0.561 (0.004, 3.998) | 0.655 | 0.71 | 0.734 (0.006, 5.341) | 0.821 | 0.821 | 1.759 (0.014, 14.082) | 0.721 | 0.721 |
| Reporter type |  |  |  |  |  |  |  |  |  |
| Health expert | Reference |  |  | Reference |  |  | Reference |  |  |
| Non-health expert | 14.399 (0.113, 105.265) | 0.189 | 0.307 | 18.307 (0.143, 137.003) | 0.163 | 0.303 | 54.030 (0.412, 468.481) | 0.085 | 0.266 |
| Missing | 13.667 (1.511, 53.824) | 0.026 | 0.067 | 17.855 (1.955, 72.345) | 0.017 | 0.066 | 16.762 (0.128, 146.482) | 0.175 | 0.36 |
| Exposure windows (days) |  |  |  |  |  |  |  |  |  |
| Nonexposure | Reference |  |  | Reference |  |  | Reference |  |  |
| 0-29 | 8.319 (3.570, 18.341) | <0.001 | <0.001 | 10.261 (4.085, 24.662) | <0.001 | <0.001 | 6.954 (1.197, 31.779) | 0.033 | 0.144 |
| 30-89 | 10.626 (4.174, 24.692) | <0.001 | <0.001 | 13.010 (4.662, 33.133) | <0.001 | <0.001 | 6.348 (0.627, 35.258) | 0.102 | 0.266 |
| 90-239 | 2.466 (0.271, 9.897) | 0.35 | 0.455 | 3.497 (0.379, 14.611) | 0.218 | 0.354 | 2.481 (0.019, 23.668) | 0.589 | 0.638 |
| 240-359 | 3.009 (0.024, 22.309) | 0.514 | 0.607 | 4.216 (0.033, 32.199) | 0.415 | 0.585 | 9.579 (0.072, 89.898) | 0.251 | 0.407 |
| ≥360 | 22.649 (7.741, 56.769) | <0.001 | <0.001 | 2.790 (0.022, 21.385) | 0.54 | 0.585 | 76.774 (21.403, 290.295) | <0.001 | <0.001 |

JADER, Japanese Adverse Drug Event Report Database; AI, angiogenesis inhibitor; aOR, adjusted odds ratio; CI, confidence interval; *q*-value, multiple-testing adjusted *P*-value; PAAD, peripheral artery aneurysm and dissection; PAA, peripheral artery aneurysm; PAD, peripheral artery dissection.

Supplementary Table 13. Multivariate Temporal Logistic Regression Analysis of AI-Associated PAAD in JADER Cohort: Subgroup Analysis

| **Characteristics** | **PAAD** | | | **PAA** | | | **PAD** | | |
| --- | --- | --- | --- | --- | --- | --- | --- | --- | --- |
| **JADER Cohort** | **aOR (95% CI)** | ***P***-**value** | ***q***-**value1** | **aOR (95% CI)** | ***P***-**value** | ***q***-**value1** | **aOR (95% CI)** | ***P***-**value** | ***q***-**value1** |
| Age group (years) |  |  |  |  |  |  |  |  |  |
| < 70 years | Reference |  |  | Reference |  |  | Reference |  |  |
| ≥ 70 years | 0.328 (0.148, 0.664) | 0.002 | 0.006 | 0.420 (0.176, 0.924) | 0.031 | 0.110 | 0.150 (0.016, 0.639) | 0.007 | 0.033 |
| Gender |  |  |  |  |  |  |  |  |  |
| Male | Reference |  |  | Reference |  |  | Reference |  |  |
| Female | 0.882 (0.467, 1.678) | 0.700 | 0.739 | 1.350 (0.633, 3.023) | 0.441 | 0.529 | 0.464 (0.132, 1.418) | 0.180 | 0.350 |
| Diabetes |  |  |  |  |  |  |  |  |  |
| No | Reference |  |  | Reference |  |  | Reference |  |  |
| Yes | 0.495 (0.099, 1.544) | 0.251 | 0.348 | 0.644 (0.127, 2.088) | 0.499 | 0.561 | 0.361 (0.003, 3.056) | 0.423 | 0.544 |
| Hypertension |  |  |  |  |  |  |  |  |  |
| No | Reference |  |  | Reference |  |  | Reference |  |  |
| Yes | 1.808 (0.872, 3.550) | 0.108 | 0.216 | 1.955 (0.831, 4.289) | 0.120 | 0.239 | 1.468 (0.359, 4.758) | 0.560 | 0.630 |
| Immunotherapy |  |  |  |  |  |  |  |  |  |
| No | Reference |  |  | Reference |  |  | Reference |  |  |
| Yes | 0.653 (0.293, 1.328) | 0.248 | 0.348 | 0.407 (0.128, 1.028) | 0.058 | 0.173 | 1.051 (0.305, 3.392) | 0.934 | 0.934 |
| Quinolones |  |  |  |  |  |  |  |  |  |
| No | Reference |  |  | Reference |  |  | Reference |  |  |
| Yes | 0.542 (0.004, 3.862) | 0.635 | 0.714 | 0.700 (0.006, 5.073) | 0.792 | 0.792 | 1.806 (0.014, 14.855) | 0.710 | 0.752 |
| Reporter type |  |  |  |  |  |  |  |  |  |
| Health expert | Reference |  |  | Reference |  |  | Reference |  |  |
| Non-health expert | 13.295 (1.461, 52.950) | 0.027 | 0.071 | 18.628 (2.038, 75.802) | 0.016 | 0.072 | 14.363 (0.108, 130.573) | 0.194 | 0.350 |
| Missing | 14.019 (0.110, 102.842) | 0.192 | 0.315 | 17.617 (0.137, 133.444) | 0.167 | 0.301 | 46.215 (0.354, 396.451) | 0.093 | 0.280 |
| Exposure windows (days) |  |  |  |  |  |  |  |  |  |
| Nonexposure | Reference |  |  | Reference |  |  | Reference |  |  |
| SMV-TKIs |  |  |  |  |  |  |  |  |  |
| 0-29 | 8.500 (2.597, 22.635) | 0.001 | 0.006 | 9.277 (2.355, 28.099) | 0.003 | 0.021 | 15.120 (2.538, 71.431) | 0.006 | 0.033 |
| 30-89 | 6.546 (1.286, 21.443) | 0.028 | 0.071 | 5.755 (0.622, 24.266) | 0.104 | 0.235 | 12.044 (1.155, 70.535) | 0.040 | 0.144 |
| 90-239 | 1.680 (0.013, 12.586) | 0.739 | 0.739 | 2.443 (0.019, 18.794) | 0.587 | 0.622 | 5.150 (0.038, 51.615) | 0.375 | 0.519 |
| 240-359 | 7.270 (0.057, 54.361) | 0.292 | 0.376 | 10.282 (0.080, 79.404) | 0.235 | 0.385 | 22.714 (0.170, 220.918) | 0.146 | 0.350 |
| ≥360 | 40.525 (12.360, 108.199) | <0.001 | <0.001 | 5.915 (0.046, 45.613) | 0.335 | 0.463 | 140.426 (34.847, 566.163) | <0.001 | <0.001 |
| anti-VMAs |  |  |  |  |  |  |  |  |  |
| 0-29 | 8.906 (3.056, 22.207) | <0.001 | 0.002 | 12.172 (4.048, 32.188) | <0.001 | <0.001 | 2.841 (0.021, 26.790) | 0.541 | 0.630 |
| 30-89 | 16.690 (5.716, 41.737) | <0.001 | <0.001 | 22.655 (7.517, 60.150) | <0.001 | <0.001 | 5.292 (0.040, 49.870) | 0.365 | 0.519 |
| 90-239 | 4.762 (0.523, 19.151) | 0.136 | 0.245 | 6.634 (0.718, 27.899) | 0.084 | 0.216 | 5.364 (0.040, 50.980) | 0.363 | 0.519 |
| 240-359 | 5.071 (0.040, 37.709) | 0.368 | 0.442 | 7.092 (0.055, 54.398) | 0.298 | 0.447 | 17.377 (0.131, 164.839) | 0.172 | 0.350 |
| ≥360 | 11.340 (1.243, 45.792) | 0.035 | 0.080 | 5.282 (0.041, 40.749) | 0.360 | 0.463 | 38.860 (3.845, 215.025) | 0.006 | 0.033 |

JADER, Japanese Adverse Drug Event Report Database; AI, angiogenesis inhibitor; aOR, adjusted odds ratio; CI, confidence interval; *q*-value, multiple-testing adjusted *P*-value; SMV-TKIs, small-molecule VEGFR tyrosine kinase inhibitors; VEGFR, vascular endothelial growth factor receptor; Anti-VMAs, anti-VEGF monoclonal antibodies; PAAD, peripheral artery aneurysm and dissection; PAA, peripheral artery aneurysm; PAD, peripheral artery dissection.

**Supplementary Figure 1. Time**-**to**-**Onset Analysis of AI**-**Related PAAD in the FAERS SMV**-**TKIs Subgroup.** (A, B) Time-to-Onset analysis of PAAD; (C, D) Time-to-Onset analysis of PAA; (E, F) Time-to-Onset analysis of PAD. FAERS, U.S. Food and Drug Administration Adverse Event Reporting System; AI, Angiogenesis Inhibitor; SMV-TKIs, small-molecule VEGFR tyrosine kinase inhibitors; VEGFR, vascular endothelial growth factor receptor; PAAD, peripheral artery aneurysm and dissection; PAA, peripheral artery aneurysm; PAD, peripheral artery dissection.

**Supplementary Figure 2. Time**-**to**-**Onset Analysis of AI**-**Related PAAD in the FAERS Anti**-**VMAs Subgroup.** (A, B) Time-to-Onset analysis of PAAD; (C, D) Time-to-Onset analysis of PAA; (E, F) Time-to-Onset analysis of PAD. FAERS, U.S. Food and Drug Administration Adverse Event Reporting System; AI, Angiogenesis Inhibitor; Anti-VMAs, anti-VEGF monoclonal antibodies; VEGF, vascular endothelial growth factor; PAAD, peripheral artery aneurysm and dissection; PAA, peripheral artery aneurysm; PAD, peripheral artery dissection.

**Supplementary Figure 3. Time**-**to**-**Onset Analysis of AI**-**Related PAAD in the JADER SMV**-**TKIs Subgroup.** (A, B) Time-to-Onset analysis of PAAD; (C, D) Time-to-Onset analysis of PAA; (E, F) Time-to-Onset analysis of PAD. JADER, Japanese Adverse Drug Event Report Database; AI, Angiogenesis Inhibitor; SMV-TKIs, small-molecule VEGFR tyrosine kinase inhibitors; VEGFR, vascular endothelial growth factor receptor; PAAD, peripheral artery aneurysm and dissection; PAA, peripheral artery aneurysm; PAD, peripheral artery dissection.

**Supplementary Figure 4. Time**-**to**-**Onset Analysis of AI**-**Related PAAD in the JADER Anti**-**VMAs Subgroup.** (A, B) Time-to-Onset analysis of PAAD; (C, D) Time-to-Onset analysis of PAA; (E, F) Time-to-Onset analysis of PAD. JADER, Japanese Adverse Drug Event Report Database; AI, Angiogenesis Inhibitor; Anti-VMAs, anti-VEGF monoclonal antibodies; VEGF, vascular endothelial growth factor; PAAD, peripheral artery aneurysm and dissection; PAA, peripheral artery aneurysm; PAD, peripheral artery dissection.
